# Transitions in chromatin conformation shaped by fatty acids and the circadian clock underlie hepatic transcriptional reorganization in obese mice

**DOI:** 10.1007/s00018-024-05364-3

**Published:** 2024-07-26

**Authors:** Ignacio Pacheco-Bernal, Fernando Becerril-Pérez, Marcia Bustamante-Zepeda, Mirna González-Suárez, Miguel A. Olmedo-Suárez, Luis Ricardo Hernández-Barrientos, Alejandro Alarcón-del-Carmen, Quetzalcoatl Escalante-Covarrubias, Lucía Mendoza-Viveros, Enrique Hernández-Lemus, Alfonso León-del-Río, Inti A. de la Rosa-Velázquez, Ricardo Orozco-Solis, Lorena Aguilar-Arnal

**Affiliations:** 1 Departamento de Biología Celular y Fisiología, Instituto de Investigaciones Biomédicas, Universidad Nacional Autónoma de México, 04510 Mexico City, Mexico; 2 Department of Computational Genomics, Centro de Ciencias de La Complejidad (C3), Instituto Nacional de Medicina Genómica (INMEGEN), Universidad Nacional Autónoma de México, Mexico City, Mexico; 3 Departamento de Medicina Genómica y Toxicología Ambiental, Programa Institucional de Cáncer de Mama, Instituto de Investigaciones Biomédicas, Universidad Nacional Autónoma de México, 04510 Mexico City, Mexico; 4 Genomics Laboratory, Red de Apoyo a la Investigación-CIC, Universidad Nacional Autónoma de México, Instituto Nacional de Ciencias Médicas y Nutrición Salvador Zubirán, 14080 Mexico City, Mexico; 5 Present Address: Next Generation Sequencing Core Facility, Helmholtz Zentrum Muenchen, Ingolstaedter Landstr 1, 85754 Neuherberg, Germany; 6 Laboratorio de Cronobiología, Metabolismo y Envejecimiento, Instituto Nacional de Medicina Genómica (INMEGEN), Mexico City, Mexico; 7 Centro de Investigacíon sobre el Envejecimiento, Centro de Investigación y de Estudios Avanzados (CIE-CINVESTAV), Mexico City, México; 8 Present Address: Instituto Potosino de Investigación Científica y Tecnológica, San Luis Potosí, Mexico

**Keywords:** Chromatin, Circadian rhythms, Genome regulatory elements, Enhancers, Chromatin conformation, Obesity, Lipid metabolism, Transcription

## Abstract

The circadian clock system coordinates metabolic, physiological, and behavioral functions across a 24-h cycle, crucial for adapting to environmental changes. Disruptions in circadian rhythms contribute to major metabolic pathologies like obesity and Type 2 diabetes. Understanding the regulatory mechanisms governing circadian control is vital for identifying therapeutic targets. It is well characterized that chromatin remodeling and 3D structure at genome regulatory elements contributes to circadian transcriptional cycles; yet the impact of rhythmic chromatin topology in metabolic disease is largely unexplored. In this study, we explore how the spatial configuration of the genome adapts to diet, rewiring circadian transcription and contributing to dysfunctional metabolism. We describe daily fluctuations in chromatin contacts between distal regulatory elements of metabolic control genes in livers from lean and obese mice and identify specific lipid-responsive regions recruiting the clock molecular machinery. Interestingly, under high-fat feeding, a distinct interactome for the clock-controlled gene *Dbp* strategically promotes the expression of distal metabolic genes including *Fgf21*. Alongside, new chromatin loops between regulatory elements from genes involved in lipid metabolism control contribute to their transcriptional activation. These enhancers are responsive to lipids through CEBPβ, counteracting the circadian repressor REVERBa. Our findings highlight the intricate coupling of circadian gene expression to a dynamic nuclear environment under high-fat feeding, supporting a temporally regulated program of gene expression and transcriptional adaptation to diet.

**Supplementary Information:**

The online version contains supplementary material available at 10.1007/s00018-024-05364-3.

## Introduction

The circadian clock system orchestrates the synchronization of metabolic, physiological, and behavioral functions in mammals over a 24-h period, enabling an efficient response to environmental changes throughout the day [1]. At the molecular level, the circadian clock operates through rhythmic transcriptional and translational loops [2]. At the core of the transcriptional activator branch operates the heterodimeric BMAL1:CLOCK (Brain and muscle Arnt-like protein-1: Circadian locomotor output cycles kaput) transcription factor, which activates the expression of a myriad of genes by binding to E-box consensus motifs in their genomic regulatory elements. These genes include their own repressors *Per* (*Period*) and *Cry* (*Cryptochrome*), along with key metabolic and cellular regulators [2, 3]. It is well established that misalignment of circadian rhythms contributes to major metabolic pathologies, including obesity, Type 2 diabetes and metabolic syndrome [4, 5]; however, the precise role of circadian clock disruption in metabolic diseases in humans remains largely elusive. Hence, identifying regulatory mechanisms involved in circadian control is of interest to find new therapeutic targets or to improve treatments for metabolic diseases.

A pivotal regulatory layer in circadian gene expression involves epigenetic control, where chromatin remodelers and epigenetic modifiers collaborate with the clock machinery to dictate robust circadian gene expression [6–8]. Distal enhancer elements are coordinately remodeled in a time-dependent manner to regulate rhythmicity and phase of transcription of target genes [9]. Enhancer function is framed in the 3D chromatin structure; hereby, the genome topology and chromatin interactions between distal regulatory elements support transcriptional cycles. For instance, in cultured mammalian cells, the clock-controlled gene *Dbp* (Albumin D-Element-Binding Protein) engages in circadian fluctuating chromosomal contacts, bringing together regulatory elements in the nuclear space that recruit the molecular clock [10]. Notably, in mouse embryonic fibroblasts and liver, the BMAL1 circadian regulator is involved in sustaining daily fluctuations in the 3D genomic architecture, promoting specific interactions between gene regulatory elements essential for rhythmic transcription of genes such as *Dbp*, *Cry1*, *Per1* or *Per2* [10–13]. Moreover, in the mouse liver, the clock repressor REVERBα (also known as NR1D1, nuclear receptor subfamily 1 group D member 1) recruits specific corepressors, including NCoR-HDAC3 (Nuclear receptor corepressors—histone deacetylase 3). This, in turn, facilitates the eviction of architectural proteins like MED1 (Mediator 1) from chromatin, contributing to the rhythmic modulation of chromatin loops underlying circadian transcription [14]. A temporally resolved genome-wide contact map in mouse liver has shown that diurnal dynamics across different genomic scales, from long-range chromatin loops to large genomic compartments, act in concert to delineate rhythmic transcription [15]. However, the participation of rhythmic chromatin topology and circadian enhancers in disease states remains largely unexplored.

Metabolic diseases and obesity emerge as a XXI Century epidemic, promoted by modern society’s lifestyles, including unhealthy intake of high calorie content meals and dysregulation of biological rhythms [16, 17]. Nutrition has a profound impact on circadian transcription in different tissues [18–20], yet the underlying regulatory mechanisms are mostly elusive. Along these lines, diet-induced obesity (DIO) triggers a wide remodeling of circadian enhancers in mouse liver, promoting rhythmic activity of specific enhancers regulating transcriptional programs of genes involved in fatty acid metabolism. This dynamic in chromatin is assisted by PPAR (Peroxisome proliferator-activated receptor) and SREBP (Sterol regulatory element-binding protein) families of transcription factors, which are crucial regulators of genes involved in de novo lipogenesis and fatty acid oxidation [21]. Additionally, activation of preexisting chromatin loops and de novo generation of loops occurs during adaptation to lipid-rich diet [22]. In this scenario, there is still a complete lack of information about (1) Whether rhythmic chromatin long-range interactions are influenced by dietary conditions and (2) how the molecular clock machinery might be involved in enhancer remodeling and de novo generation of loops in response to lipid-rich diets. Hereby, understanding on how chromatin loops are remodeled during the circadian cycle in response to high-fat diet and their impact in transcriptional rewiring is needed for delineating the role of the circadian clock in dysfunctional metabolism and physiology.

To approach these gaps of information, we explored the daily fluctuations on chromatin contacts between distal regulatory elements of key metabolic control genes—*Pparα*, *Pparγ2*, and *Serbp1c*—and the clock-controlled gene *Dbp* in the livers of both lean and obese mice. We demonstrate that specific lipid-responsive regulatory regions recruit core-clock transcription factors in conjunction with transcriptional regulators participating in lipid metabolism control. Notably, for the *Dbp* gene, a new chromatin interactome was detected in fatty livers, which did not significantly alter *Dbp* expression; rather, distal interactions were oriented to promote expression of metabolic genes, including *Fgf21* (Fibroblast growth factor 21), *Akt1s1* (AKT1 substrate 1) or *Irf3* (Interferon regulatory factor 3). In contrast, *Pparα*, *Pparγ2* and *Srebp1c* exhibit increased interactions with regulatory elements under high-fat feeding, leading to their transcriptional activation. Remarkably, we found regulatory elements responsive to lipids through their activation by the transcription factor CEBPβ (CCAAT enhancer binding protein beta), which counteracts the activity of the circadian repressor REVERBα. Together, our results highlight the sophisticated coupling of circadian gene expression to a dynamic nuclear environment under high fat feeding, supporting a temporally regulated program of gene expression and transcriptional adaptation to diet.

## Materials and methods

### Experimental model

Male C57BL/6 J mice were housed under 12 h light:12 h dark (LD12:12) with food and water available ad libitum. 7–8 weeks old mice were randomly distributed in two groups, and were fed for 12 weeks with either a chow diet (CD group, 18% calories from fat; 2018S Tekland, ENVIGO) or a high-fat diet (HF group, 53% calories from fat; TD.160547 Tekland, ENVIGO). Mice were sacrificed either at ZT6 or ZT18 (n = 6 mice per time point and diet), and livers were snap frozen and kept at -80ºC for further processing. Animal care was in accordance with the Internal Committee for the Care and Use of Laboratory Animals (CICUAL) at the Instituto de Investigaciones Biomédicas, protocol number ID230 (UNAM, Mexico).

### Glucose and insulin tolerance tests

At 10 and 11 weeks of feeding paradigm, mice were subjected to a glucose tolerance and insulin tolerance tests (GTT / ITT). For the GTT, mice were fasted for 12 h, and injected intraperitoneally (IP) with D-glucose (G7021, SIGMA) at 2 mg/kg. For the ITT, insulin (HI0210, Eli Lilly) at 0.6 U/kg was IP injected 4 h post fasting. Circulating glucose was measured from tail using a glucometer (ACCU-CHEK, ROCHE) at 0, 15, 30, 60, 90 and 120 min after IP injection. Both procedures were performed at ZT1 and ZT13, with n = 6 mice per group.

### Oil red O staining

Frozen livers were embedded in OCT medium (Tissue-Tek, 4583) and cut into 10-μm sections using a Leica cryostat. Tissue sections were fixed in slides with 4% paraformaldehyde for 2 min and immediately washed twice with deionized water. Oil Red O (SIGMA, O1391) working solution (3.75 mg/ml in 60% isopropanol) was applied on slides for 5 min at room temperature (RT) and washed with abundant deionized water to eliminate colorant excess. The images were documented with Axiocam EEc 5 s camera coupled to a ZEISS Primovert microscope, using a 40X magnification.

### RNA extraction and cDNA synthesis

60 mg of tissue were homogenized (Benchmark Scientific, D1000 homogenizer) for 30 s with 0.75 ml of Trizol^™^ (15,596,018, Invitrogen). Homogenates were incubated for 5 min at room temperature (RT), then 0.15 ml of chloroform was added, shaked and incubated at RT for 3 min followed by centrifugation for 15 min at 13,000 rpm at 4 °C. The aqueous phase was recollected, and 0.4 ml of isopropanol was added. After a 10 min incubation at RT, RNA was precipitated by centrifugation for 20 min at 13,000 rpm and 4 °C. RNA was washed twice with 0.75 ml of 75% ethanol and resuspended in 50 µl of molecular biology grade water. For cDNA synthesis, 1 μg of RNA was retrotranscribed with SuperScript^™^ III RT (Invitrogen, 18,080,093) according to manufacturer’s instructions, using 100 ng of random hexamers (Invitrogen, N8080127) per sample.

### Real-time PCR

RT-qPCR reactions were performed with 10 ng of cDNA using the iTaq Universal SYBR^®^ Green Mix (1,725,121, BioRad) in a final volume of 10 µl. A CFX96 Touch Real-Time PCR Detection System (BioRad) was used with the following program: 30 s at 95 °C, followed by 40 cycles of 5 s at 95 °C, 30 s at 60 °C. Single-product amplification was verified by an integrated post-run melting curve analysis. Values were normalized to the housekeeping genes *Gapdh* and *B2m*. The geometric mean was used to calculate Ct values of the housekeeping genes and expression values for the genes of interest were determined using ΔCT methodology. Primers are listed in Supplementary Table S1.

### 4C-seq

Baits were defined in regions with regulatory potential for each gene as follows: *Dbp* bait was located in the intron 2 of *Dbp* gene, containing two E-boxes essential for rhythmic expression of the gene as described in [10], *Ppara* and *Pparg2* baits were located in their promoter regions, and *Srebp1c* bait was designed within intron 1, which shows strong regulatory potential with high H3K27ac levels in mouse liver. Two livers per dietary condition and time point were randomly selected for 4C experiments**.** 4C templates were prepared from 0.5 to 0.7 g of liver tissues homogenized in PBS 1X pH 7.4, filtered in 70 µm cell strainer and diluted to 30 ml with PBS. Cells were pelleted and crosslinked in 25 ml of DMEM with 1% of formaldehyde at room temperature under rotation for 15 min and the reaction was immediately quenched with glycine at 0.125 M final concentration for 5 min at 4 °C. Nuclear isolation was performed in NI buffer (10 mM Tris HCl, 10 mM NaCl, 0.2% NP-40) for 1 h at 4 °C. For first restriction, selected six-cutter enzymes were EcoRI for *Pparγ2* bait, and HindIII for *Dbp*, *Pparα* and *Srebp1c* baits. Ten million nuclei per sample were resuspended in 500 µl of 1X NEBbuffer 2.1 (New England BioLabs) containing 0.3% SDS and incubated 10 min at 60 °C and 50 min at 37 °C. Triton X100 was added at 1.8% v/v, incubated for 1 h at 37 °C, and 400 U of restriction enzyme were added and incubated at 37 °C overnight with agitation. First ligation was performed with 100 U of T4 DNA ligase (ThermoScientific Cat. No. EL0011) with 5% Polyethylene glycol in 7 ml final volume, for 6 h at 16 °C. DNA crosslinking was reversed by adding 30 µl of 10 mg/ml proteinase K (EMD Millipore Cat. No. 124568) and incubating for 6 h at 65 °C, followed by addition of 30 µl of 10 mg/ml RNAse A (Sigma Cat. No.R5000) and incubation for 45 min at 37 °C. DNA was purified with phenol/chloroform extraction and sodium acetate/ethanol precipitation.

The purified DNA had a second restriction with 200 U of Csp6I (New England BioLabs) in 500 µl reaction volume. Finally, a second round of ligation with 200 U of T4 DNA ligase with 5% PEG in a 14 ml final volume was performed. Circular DNA was resuspended in molecular grade H2O. Efficiency of each reaction was evaluated by loading DNA in 0.8% agarose gel.

Libraries were prepared with a two-step PCR procedure. For each 4C template, eight reactions of 100–200 ng were amplified by PCR using high fidelity Hot Start Phusion II DNA polymerase (ThermoFisher, F549) and long-template specific-bait primers containing the NexteraXT overhang sequences (Supplementary Table S1), for 2 min at 98 °C, (15 s at 98 °C, 1 min at 48 °C, 3 min at 72 °C) × 30 cycles and a final extension of 7 min at 72 °C. PCRs were pooled to serve as template for a subsequent PCR using NexteraXT index kit. PCR products were size selected with 2% agarose gel to 200 bp-2 kb, purified with gel purification kit (Qiagen) and fragment size distribution analyzed with bioanalyzer tapestation (Agilent).

Libraries were sequenced with Illumina HiSeq platform, in *Dbp* and *Pparγ2* cases using single end 100-bp read length, meanwhile *Pparα* and *Srebp1c* libraries were sequenced with paired-end 150-bp read length. Two biological samples per condition were analyzed.

### 4C-seq analysis

Raw data quality was evaluated with FastQC. Adapters and primer sequences were trimmed using *Trim Galore*, and files were aligned to mm9 mouse genome with *bowtie2*. Close *cis* interactions were identified using the FourCSeq pipeline in *R* [23]. Briefly, Bam files were aligned to a in silico fragmented genome with the corresponding restriction enzymes (HindIII/Csp6I or EcoRI/Csp6I) and normalized counts (RPKM) for every restriction fragment were obtained. The first two fragments upstream and downstream from each bait were not considered for analysis. Count values were transformed with a variance stabilizing transformation and z-scores were calculated, considering as significant interactions those with a z-score ≥ 2.0 and an FDR ≤ 0.05 in both replicates. In the particular case of the *Srebp1c* bait, a z-score ≥ 1.5 and an FDR ≤ 0.05 were considered as threshold. For analysis of differential interactions between conditions, we calculated an average z-score ratio, setting as threshold a ratio ≥ 1.5, while keeping an FDR ≤ 0.05.

### Analyses of the trans contacts from 4C-seq

RPKM bedgraph files were used as input for the 4Cker program[24], with n = 2 and k = 20, using the *transAnalysis* function. Coordinates defining the interactions for each bait were visualized using RCircos package[25]. These analyses were performed in *R*.

### Identification and visualization of TADs from mouse liver

Previously processed HiC data from mouse liver was obtained from GSE104129 [14]. These HiC matrix files were further processed with the Galaxy HiCExplorer version [26]. HiC.matrix files were converted to.cool files with *hicConvertFormat* tool and plotted with *hicPlotTADs* tool. Matrices were visualized together with the TAD and subTAD coordinates in BED files downloaded from GSE104129, and with the obtained tracks with 4C-seq signal and significant interactions. The method for calling TADs and SubTADs is described in Ref. [14], and is based on the directionality index method as detailed in Ref. [27]. A tolerance distance of 200 kbps was applied to determine overlap.

### Western blot

30–50 mg of liver tissues were lysed with cold RIPA buffer (1 mM DTT, 10 mM Tris–HCl, 30% glycerol, 1 mM EDTA, 1% Triton X-100, 1 mM Na3VO4, 1 mM PMSF, 15 mM sodium azide and 1:25 v/v cOmplete ROCHE). Homogenates were centrifuged at 19,000 × g for 30 min at 4 °C, and supernatants were recovered. Protein content was determined with Bradford method. 30 µg of protein per sample were separated in SDS PAGE, using a 7% acrylamide gel to allow for detection of differences in BMAL1 phosphorylated states, and transferred to PVDF membranes. Membranes were blocked with 5% w/v non-fat milk in 0.05% PBST for one hour and hybridized with the corresponding primary antibodies overnight at 4 °C. Membranes were washed three times with PBST and incubated with the corresponding secondary antibody for 1 h at RT, followed by three washes with PBST. For specific band visualization, the Immobilon Western Chemiluminiscent HRP Substrate (Millipore, WBKLS0100) was used, and signals were recorded in a Gel Logic 1500 Imaging System (KODAK). Protein bands were quantified by densitometric analysis using ImageJ software. Relative densitometric units were calculated by dividing the densitometric band value of protein of interest by the GAPDH protein for each sample, and then normalized to CD ZT6 condition unless otherwise specified in the figure caption. Primary antibodies used in this study were: PPARY (2443) and REVERBA (13,418), both at 1:1000 dilution, from Cell Signaling; CEBPA (365,318) and CEBPB (7962), 1:500, from Santa Cruz Biotechnology; BMAL1 (93,806), 1:2000, from Abcam; PER2 (Per21A), 1:1000, from Alpha Diagnostics; CRY1 (A302-614A), 1:2000, from Bethyl laboratories; GAPDH (GT-239), 1:20,000, from GeneTex; TUBULIN (T5168), 1:8000, from Sigma. Secondary antibodies were from Sigma, anti-rabbit IgG (A0545, 1:10,000 for PPARY, 1:80,000 for CRY1, REVERBA and PER2, 1:150,000 for BMAL1) and anti-mouse (A09044, 1:20,000 for CEBPA and CEBPB, 1:80,000 for TUBULIN).

### Enhancer selection and analyses

Enhancer candidates were considered as regions colocalizing with the interactions detected by 4C-seq +–5 kb with enrichment in genomic features associated with active enhancers, as H3K27ac, chromatin accessibility measured with ATAC and FAIRE-seq (see data availability section). Also, as a plus feature, some of these regions colocalized with annotated regulatory regions by ENCODE [28]. Genomic coordinates of candidates are indicated in Supplementary Table S3.

CistromeDB Toolkit [29] was used to search for TFs defined to specific coordinates in the mm10 version of the mouse genome. ChIP-seq data were filtered for liver and adipose tissue TFs in defined intervals according to genomic coordinates of enhancer sequences. Over-represented TFs were detected as a measure of overlapped peaks in the regions of interest.

The enhancer sequences were compared to the human genome (hg38) using NCBI nucleotide blast tool [30], searching for very similar regions. Percentage of conservation and genomic coordinates in human were obtained.

### Cloning of enhancers

Enhancer sequences were cloned in pGL4.23 plasmid (Promega) upstream of the minimal promoter using KpnI (ThermoFisher Scientific, ER0501) and HindIII (New England Biolabs, R0104S) restriction enzymes. Briefly, 10 µg of vector were digested with 30 U of each enzyme in 70 µl of reaction mix in NEBuffer TM 2.1 1X (New England Biolabs, B27202S) for 6 h at 37 °C. Reaction was inactivated at 80 °C for 10 min. Digested vector was recovered by purification on a 0.7% agarose gel using QIAquick Gel Extraction kit (QIAGEN, 28,704). Enhancer sequences were amplified from mouse genomic DNA using specific primers flanked by the restriction enzymes recognition sequences (Supplementary Table 1). Each insert was amplified with the high fidelity Phusion Hot Start II DNA Polymerase (ThermoFisher Scientific, F549S). DNA was recovered by purification on a 1.0% agarose gel using QIAquick Gel Extraction kit (QIAGEN, 28,704). Inserts were digested with 10 U of each enzyme in 50 µl of reaction mix containing NEBuffer TM 2.1 1X (NEB, B27202S) for 4 h at 37 °C and inactivation at 80 °C for 10 min. DNA was cleaned with MinElute Reaction Cleanup Kit (QIAGEN, 28,204) and eluted in molecular grade water. Ligations were performed using 50 ng of digested vector in a 1:10 vector:insert molar proportion, with 5U of T4 DNA ligase (ThermoFisher Scientific, EL001) in 10 µl of volume reaction at 16 °C overnight and inactivation of ligase at 65 °C for 10 min. Constructions were cloned in TOP10 E coli, and DNA was purified with E.Z.N.A Plasmid Maxi Kit (Omega BIO-TEK, D6922). Successful cloning was verified by Sanger sequencing.

### Cell culture and treatments

AML12 cell line (ATCC, CRL-2254) was cultured in DMEM-F12 medium (Gibco, 12,400–029) supplemented with 10% fetal bovine serum (FBS), ITS 0.5X (5 µg/ml insulin, 2.75 µg/ml transferrin, 2.5 ng/ml selenium; Gibco, 41,400,045), Penicillin/Streptomycin 1X (Biowest, MS00P01014) and 40 ng/ml of dexamethasone (Sigma, D4902). For treatment with a mixture of palmitate/oleate, 12,500 cells were seeded in 24 well plates in the complete medium with 5% FBS. After 48 h, cells were treated with 0.25 mM of palmitate (Sigma, P0500)/oleate (Sigma, O1008) mixture in a 1:2 proportion for 48 h prior to transfection. Cells treated with 1% of free fatty acid albumin (FFA-BSA) were used as control.

Palmitate/Oleate fatty acids were solubilized using alkaline NaOH 0.1 N at 80 °C to generate the corresponding salt and complexing with FFA-BSA in a 6:1 proportion FA/FFA-BSA for 1 h at 37 °C in 0.9% NaCl solution with constant agitation at 1000 rpm. ORO staining of cells was performed following our previously described protocols [31].

### Enhancer activity measured by luciferase reporter assay

AML12 cells seeded in 24-well plates were transfected with 200 ng of enhancers fused to the luciferase vector, 200 ng of *LacZ* reporter, 100 ng myc–CLOCK–pCDNA3, 200 ng myc–BMAL1–pCDNA3, which have been described previously[32]. pCDNA3 vector was used to adjust DNA quantity. DNA was transfected using BioT transfection reagent (Bioland Scientific LCC, B01-00) in a proportion 1.5:1 BioT:DNA, except for Fig. 5F, where Lipofectemine 2000 (Thermo Fisher) was used to a 2:1 ratio. The total amount of applied DNA per well was adjusted by adding pcDNA3 vector. Cells were allowed to recover for 24 h more and luciferase assay was performed. Lipid treatment was maintained during transfection and until the end of the protocol.

Cells were washed with 700 µl of PBS 1X and lysed in 100 µl of lysis buffer (25 mM Tris–phosphate pH 7.8, 2 mM EDTA, 1 mM DTT, 10% glycerol, 1% TritonX-100) for 20 min at room temperature with vigorous orbital agitation, followed by subsequent incubation at -80 °C for 40 min. Supernatants were recovered for luciferase assay. 20 µl of lysate were added to a 96-well luciferase plate, and 100 µl of luciferase reaction buffer (20 mM Tris–phosphate pH 7.8, 1.07 mM MgCl_2_, 2.7 mM MgSO_4_, 0.1 mM EDTA, 33.3 mM DTT, 470 µM luciferin, 530 µM ATP, 270 µM Coenzyme-A) were subsequently added to the lysates. Luminescence was measured using a Synergy H1 (BioTek) multi-mode reader, with 1 s of integration time.

For β-galactosidase assay, 40 µl of lysate were assessed in a 96 well plate containing 120 µl of tampon Z (60 mM NaHPO_4_, 40 mM NaH_2_PO_4_-H_2_O, 10 mM KCl, 1 mM MgSO_4_, 0.7 mg/ml ONPG and 3.50 µl/ml β-mercaptoethanol). The plate was incubated 37 °C until the yellow color appeared, and the reaction was stopped with 50 µl of 1 M Na_2_CO_3_. β-galactosidase activity was measured at 450 nm using a Synergy H1 (BioTek) multi-mode reader. Transfection efficiency was normalized by dividing luciferase signal by the β-galactosidase signal.

Plasmids expressing BMAL1 and CLOCK have been previously described[32] and were a kind gift from Paolo Sassone-Corsi. Plasmids expressing REVERBβ CEBPβ and A-CEBPβ were purchased from Addgene, Cat. No. 22745 gifted by Bruce Spiegelman and described in [33], Cat. No. 49198 gifted by Jed Friedman and Cat. No. 33363 gifted by Charles Vinson respectively.

### Chromatin immunoprecipitation (ChIP)

ChIP experiments were performed as previously described [34]. Briefly, 200 mg of liver tissue were homogenized in PBS. Dual crosslinking was performed in a final volume of 1 ml using 2 mM of DSG (Disuccinimidyl glutarate, ProteoChem, CAS: 79,642–50-5) for 10 min at RT on a rotary shaker. DSG was washed out and 1% formaldehyde (Sigma-Aldrich, F8775) in PBS was added an incubated for 15 min at RT with rotataion. Crosslinking was topped with 0.125 M glycine for 5 min on ice. Two washes with ice-cold PBS were performed, and nuclei were isolated in 600 μL of ice-cold nuclei preparation buffer (NPB: 10 mM HEPES, 10 mM KCl,1.5 mM MgCl2, 250 mM sucrose, 0.1% IGEPAL CA-630) at 4 °C for 5 min in rotation. Nuclei were collected by centrifugation at 1,500 g for 12 min at 4 °C and, and resuspended in 600 μL of cold nuclear lysis buffer (10 mM Tris pH 8, 1 mM EDTA, 0.5mMEGTA, 0.3% SDS, 1 × cOmplete™ Protease Inhibitor Cocktail, Roche) for 30 min on ice. 300 μL of lysates were sonicated using a Bioruptor Pico Sonicator (Diagenode) for 15 cycles (30 s ON/30 s OFF). Chromatin fragments (100–500 bp) were evaluated on agarose gels using 10 μL of sonicated chromatin for DNA purification using the phenol method. 600 μL of ice-cold ChIP-dilution buffer (1% Triton X-100, 2 mM EDTA, 20 mM Tris pH 8, 150 mM NaCl, 1 mM PMSF, 1 × cOmplete™ Protease Inhibitor Cocktail, Roche) was added to the chromatin, and 10% volume was recovered as the Input. Immunoprecipitations were set overnight at 4 °C, by adding 20 μL of magnetic beads (Cat. No. #16–662, Sigma-Aldrich) and the antibodies: 8 μl Rev-Erbα (E1Y6D) Rabbit mAb (Cell Signaling Cat. No. #13,418) or 3 μl C/EBP beta (H-7) mAb (Santa Cruz Cat. No. sc-7962), in 900 μL final volume. Immunoprecipitations with 3 μL of IgG (Sigma-Aldrich, Cat. No. 18765) were done simultaneously. Sequential washes of the magnetic beads were performed for 10 min at 4 °C, as follows: Wash buffer 1 (20 mM Tris pH 8, 0.1% SDS, 1% Triton X-100, 150 mM NaCl, 2 mM EDTA),Wash buffer 2 (20mMTris pH 8, 0.1% SDS, 1% Triton X-100, 500 mM NaCl, 2 mM EDTA), Wash buffer 3 (10 mM Tris pH 8, 250 mM LiCl, 1% IGEPAL CA-630, 1% sodium deoxycolate) and TE buffer (10 mM Tris pH 8, 1 mM EDTA). To isolate immunoprecipitated DNA, 400 μL of fresh elution buffer (10 mM Tris pH 8, 0.5% SDS, 300 mM NaCl, 5 mM EDTA, 0.05 mg/mL proteinase K) was added to the magnetic beads followed by incubation overnight at 65 °C. A treatment with RNase A at 0.1 mg/ml for 30 min at 37 °C was performed. The DNA was purified from the IPs and Inputs by the phenol-based method, and DNA was precipitated with ethanol in the presence of 20 μl glycogen (10,901,393,001, Roche) at − 80 °C overnight, and resuspended in 40 μl of MQ water. qRT-PCR reactions were set using primers described in Supplementary Table S1.

### HiC contact matrix

Normalized ICED HiC matrices corresponding to ZT10 in the WT and liver-specific *Rev-erbα* KO conditions were obtained from Kim et al., 2018 [14] (GSE104129). The HiCExplorer [35] function *hicConvertFormat* was used to convert the matrices to cool format. Subsequently, the Log_2_ ratio between the WT and KO contact matrices was calculated and plots were generated using the *hicCompareMatrices* function. Increased interactions were defined as described in REF. [14].

### GRO-seq and microarray expression data analyses

Gro-seq data were obtained from GSM1437746 which were analyzed as described [9]. Briefly, uniquely mapped reads were extended to 150 bp in the 5’ to 3’ direction, transcription tag counts were normalized by Reads Per Kilobase of transcript per Million (RPKM) and Bigwig files were generated using HOMER v4.3 [36] and visualized in the WashU epigenome browser [37] in the strand where the gene is transcribed. Gene body transcription level was calculated and plotted by counting reads beginning at the TSS. Microarray expression data form livers from WT and *RevErb*α liver-specific KO mice were obtained from GSE59460 and analyzed using GEO2R, which uses limma to perform differential expression analysis and to calculate *p* value [38].

### Statistical analyses

Analyses were performed using GraphPad Prism V8. Data are presented as the mean +–standard error, using one-way or two-way analysis of variance (ANOVA) followed by Tukey´s posttest, unless otherwise indicated. When two-way ANOVA was used, the independence between the factors (e.g., time and diet, or plasmid and treatment) was tested and reported using the “interaction” term. Differences between groups were considered as statistically significant with a *P* < 0.05.

## Results

### Daily transitions in long-range chromatin interactions in mouse liver are defined by the type of diet

We implemented a mouse model of diet-induced obesity by feeding mice with either a high fat diet (60% Kcal from fat, HF group) or a chow diet (CD group) for 12 weeks (See methods section). Significantly increased body weight was observed in the HF group compared to controls (Figure S1A, *P* < 0.001, Two-way ANOVA followed by Tukey posttest). As expected, obese mice showed impaired glucose tolerance (Figure S1B, *P* < 0.001, One-way ANOVA followed by Tukey posttest) and displayed poor ability to lower circulating glucose levels upon insulin injection (Figure S1C; *P* < 0.001, One-way ANOVA followed by Tukey posttest), evidencing abnormal high glucose levels and systemic insulin resistance associated to HF-diet feeding. This effect was more pronounced at the onset of the rest phase (ZT1; ZT is Zeitgeber time, where light is on at ZT0 and off at ZT12), coincident with previous reports showing that insulin sensitivity follows a circadian rhythm [39]. Because hepatic lipid deposition is a major feature of insulin resistance in the liver, we investigated hepatic steatosis at the macroscopic level, showing that HF-fed mice developed hepatomegaly and a yellow-orange liver coloration (Figure S1D). Subsequent ORO staining confirmed hepatic lipid accumulation in HF-fed mice (Figure S1D). Then, we analyzed expression of circadian genes at two representative time points, ZT6 (day-time) and ZT18 (night-time). As expected, the expression of *Bmal1*, *Reverbα*, *Cry1*, and *Per2* genes showed significant fluctuations between ZT6 and ZT18 (Figure S1E; P < 0.01, Two-way ANOVA with Bonferroni post-test). At the protein level, REVERBα and PER2 also fluctuated between ZT6 and ZT18. Although these two time points do not capture CRY1 variations in the livers of CD-fed mice, CRY1 was significantly increased at ZT18 in the livers of obese mice due to its advanced phase under these conditions [20, 34, 40, 41]. A similar pattern was observed for BMAL1 phosphorylation (Figure S1F). Together, these data demonstrate a functional circadian machinery in the liver of these cohort of mice and indicate that our mice fed a HF diet developed a metabolic phenotype with obesity, insulin resistance and hepatic steatosis, commonly associated to type 2 diabetes.

CLOCK:BMAL1 are pioneering transcription factors for rhythmic genes, and their disfunction (i.e., through genetic or pharmacologic interventions) has been associated with metabolic disorders, including fatty liver disease [42]. Bmal1 deficiency reduces hepatic insulin response, opposing AKT phosphorylation and decreasing de novo lipogenesis [43]. During high-fat feeding, hepatic *Bmal1* deficiency results in marked insulin resistance and liver steatosis [44]. Because BMAL1 participates in delineating chromatin loops for rhythmic transcription, it is possible that rhythmic interactions are altered in the liver of obese mice promoting, at least to some extent, hepatic metabolic derangements. Hereby, we applied Chromatin Conformation Capture followed by sequencing (4C-seq) to define long range chromatin interactions in livers from control diet and HF-fed mice. The 4C-seq technique was designed to detect interactions between a selected locus, also known as “bait” or “viewpoint”, and other genomic regions, thus providing highly specific and spatially resolved contact profiles around the bait [45–47]. As bait, we first selected the *Dbp* gene, based on our previous studies describing that this locus engages in rhythmic chromatin interactions during the circadian cycle which depend on BMAL1 in mouse embryonic fibroblasts [10]. Additionally, we sought to explore the long-range interactions from viewpoints located within genes participating in the metabolic phenotype activated by diet, including *Ppar*γ, *Ppar*α and *Srebp1c*. Specifically, the reorganization in circadian gene expression in mouse fatty liver is largely directed by the coordinated action between the transcription factors PPARγ, PPARα and SREBP, through the sequential activation of their targets along the day [21, 40]. Hereby these genes constitute important objectives to investigate, as their regulation at the chromatin level in response to lipids might underlie a large part of the transcriptional reprogramming.

**Fig. 1:**
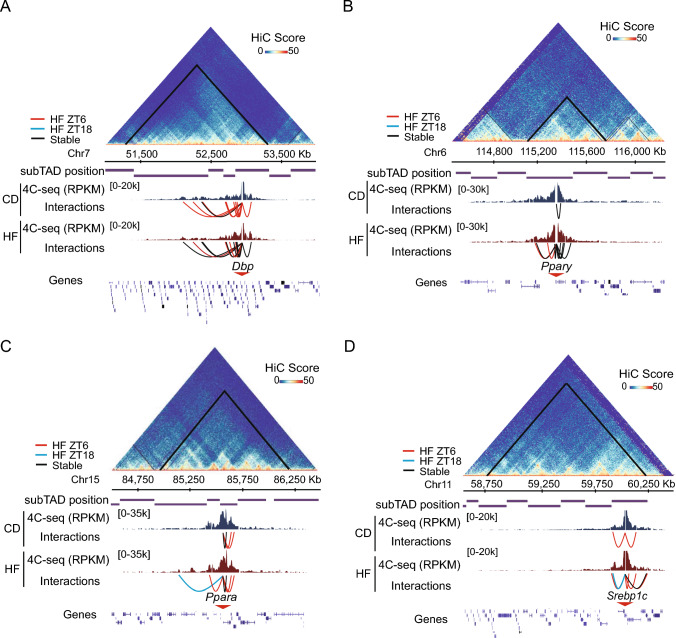
Rhythmic and specific chromatin reorganization in mouse liver under nutritional challenge. **A**–**D** 4C-seq data for genes *Dbp* (**A**), *Ppary2* (**B**), *Ppara* (**C**) and *Srebp1c* (**D**) is shown within the TAD allocating the bait. For each selected gene, the HiC data from mouse liver is plotted as a heatmap depicting frequencies of interactions (HiC Score), and TAD position is indicated (black lines within the HiC contact matrix), and subTADs are delineated. Underneath, the 4C-seq data from mouse liver (n = 2) fed either control diet (CD) or high fat diet (HF) is presented in Reads per Kilobase per Million (RPKM). Spider plots represent statistically significant contacts (FDR ≤ 0.05) at ZT6 (red), ZT18 (blue) or at both time points (black, stable contacts). The genomic positions in mm10 coordinates and gene density are indicated on the horizontal axis

We performed 4C-seq analyses on these genomic loci as previously described [10]. For each bait, we obtained sequences of adequate quality for the robust characterization of their physical interactions [48], with more than 40% of the reads mapped to the chromosome allocating the selected locus (Supplementary Table S2). To call specific interactions, we adopted the FourCSeq package [23] to calculate a *Z*-score for each restriction fragment, representative of the number of standard deviations by which the reads for a fragment is distinct from the fitted contact decay trend, from fragments within 2 Mb around the bait, and calculated their associated *P*-values (FDR < 0.05, see methods section). This analysis showed specific long-range interactions that overlapped between replicates for *Dbp*, *Ppar*γ, *Ppar*α and *Srebp1c* loci, which were restricted to their respective hepatic topologically associated domain (TAD) (Fig. 1A-D), consistent with the notion that TADs provide spatial insulation for dynamic interactions between regulatory regions [27, 49–51].

In mouse liver, two main phases define alternate chromatin recruitment of circadian transcriptional activators and repressors, centered around the circadian times ZT6 and ZT18 respectively [7, 52]. Hence, rhythmic positioning of clock proteins underlies chromatin transitions at the regulatory elements of clock-controlled genes [53]. Hereby, these two time points were selected to profile chromatin interactions. Differences in contact frequencies between distinct conditions were calculated using DESeq2 methodology [23, 54], calling differential contacts when *P*adj. < 0.05 (Wald test). As expected, for each bait we found architectural variations depending on the ZT [10, 14, 15] illustrated for example by increased interactions at ZT6 vs ZT18 for the *Dbp* and Pparγ2 baits (Fig. 1A–D). Interestingly, for all selected baits, rhythmic chromatin interactions were extensively reshaped in HF fed mice when compared with the control mice. This reorganization was also apparent for the interactions in *trans* (Figure S2A–D), some of which were previously described to be rhythmic for the *Dbp* promoter in mouse embryonic fibroblasts [10].

Hereby, we found a specific rhythmic interactome for each nutritional condition, with a diet-associated reorganization of long-range interactions whose extent appeared markedly gene-specific [6, 22]. We then turned our focus to understand the function of differential chromatin landscapes depending on time-of-day and diet.

### Diet-induced chromatin contacts between regulatory regions shape the transcriptional landscape in livers from obese mice

**Fig. 2:**
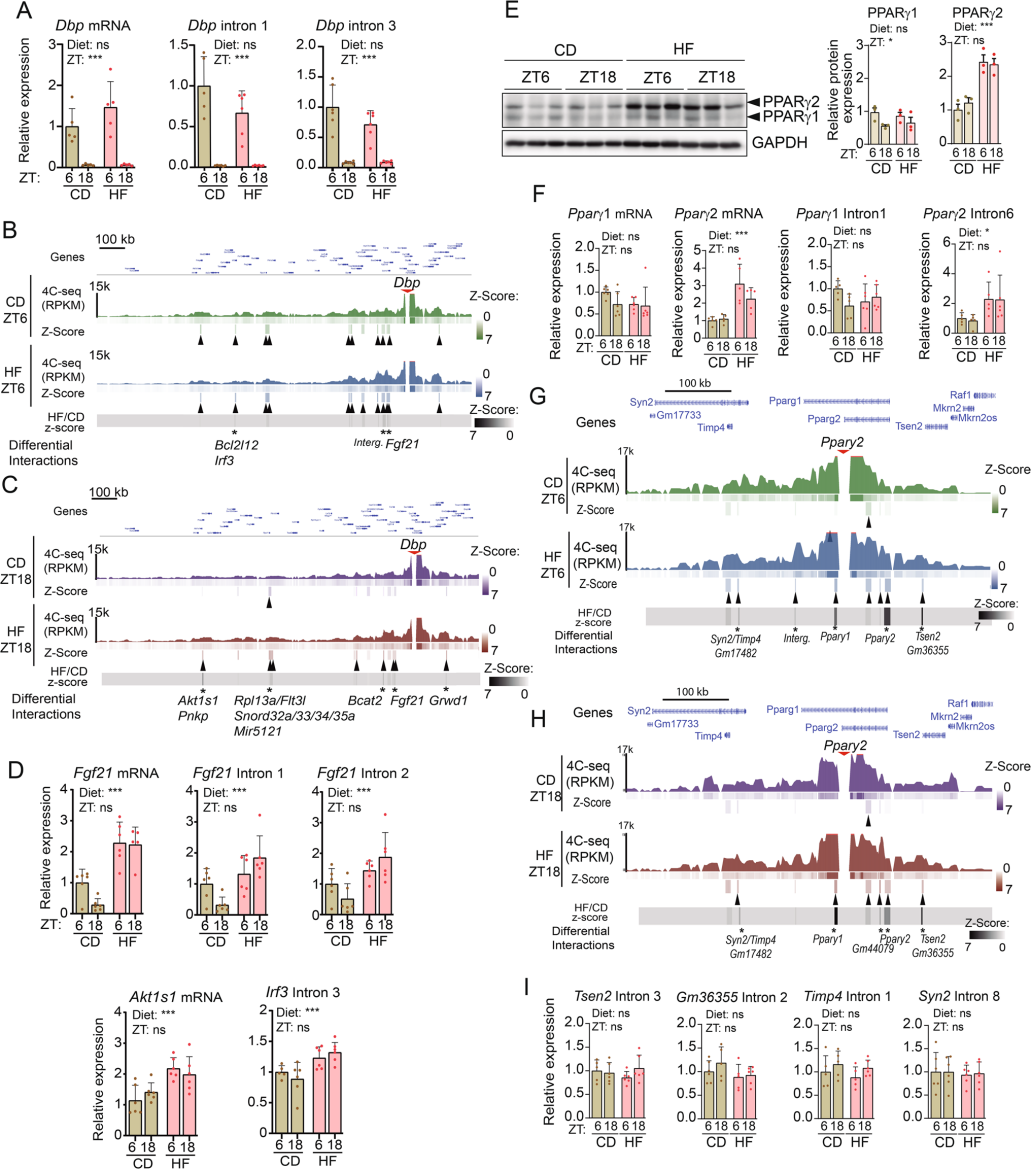
Chromatin topology around the day is shaped by diet. **A** Histograms show quantification through RT-qPCR of mature (*Dbp* mRNA) or nascent (*Dbp* intron 1, *Dbp* intron 2) *Dbp* transcript from mouse liver fed either a control (CD) or a high fat (HF) diet, at two circadian times (ZT6 and ZT18). n = 5–6 biological replicates. **B**, **C** *Dbp* interactome in a 2 Mb genomic window in CD and HF groups at ZT6 (B) and ZT18 (**C**). Interactions with *z*-score ≥ 2 and FDR ≤ 0.05 are indicated as black arrowheads. Differential contacts between HF and CD with a (HF/CD *z*-score rate ≥ 1.5) are indicated with *. Genes allocated within contacts are indicated. **D** RT-qPCR of mature (*Fgf21* mRNA, *Akt1s1* mRNA) or nascent (*Fgf21* intron 1, *Fgf21* intron 2, *Irf3* Intron 3) transcripts from mouse liver fed either a control (CD) or a high fat (HF) diet, at two circadian times (ZT6 and ZT18). n = 6 biological replicates. **E** PPARγ1 and PPARγ2 protein expression in mouse liver fed either control (CD) or high fat (HF) diet was assessed by western blot at two circadian time points (ZT6 and ZT18). Histograms represent quantification of 3 independent biological replicates, normalized to GAPDH**. F)** RT-qPCR of mature (*Pparγ1* mRNA, *Pparγ2* mRNA) or nascent (*Pparg1* intron 1, *Pparg2* intron 6) transcripts from mouse liver fed either a control (CD) or a high fat (HF) diet, at two circadian times (ZT6 and ZT18). n = 6 biological replicates. **G**, **H** *Pparγ2* interactome in a 2 Mb genomic window in CD and HF groups at ZT6 (**G**) and ZT18 (**H**). Interactions with *z*-score ≥ 2 and FDR ≤ 0.05 are indicated as black arrowheads. Differential contacts between HF and CD with a (HF/CD *z*-score rate ≥ 1.5) are denoted with *. Genes allocated within contacts are indicated. **I** RT-qPCR of the indicated nascent transcripts from mouse liver fed either a control (CD) or a high fat (HF) diet, at two circadian times (ZT6 and ZT18). n = 6 biological replicates. For RT-qPCR analyses, expression of *B2m* and *Tbp* housekeeping genes was used to normalize, and data from CD at ZT6 was set to 1. Two-way ANOVA was applied for statistical analyses. *p < 0.05, **p < 0.01; ***p < 0.001. Histograms represent mean ± standard error. Interg.: Intergenic region

First, we focused on *Dbp,* a clock-controlled gene whose expression in mouse liver is largely driven by CLOCK/BMAL1 rhythmic recruitment to E-boxes within the promoter and introns [55]. As expected, *Dbp* expression was higher at ZT6, and silenced at ZT18 both at the mRNA and pre-mRNA levels [55] (Fig. 2A). As we previously reported in MEFs [10], time-dependent chromatin interactions of *Dbp* locus were detected also in livers from control mice (Fig. 2B, C, upper panels, black arrowheads). Coincident with previous reports in mouse liver [15], at ZT6, the time of *Dbp* maximal transcriptional output, 10 significant long-range chromatin interactions were detected, while just one remained at ZT18, when *Dbp* expression is lowest. Unexpectedly, *Dbp* promoter in livers from obese mice engaged in novel contacts specifically at ZT18, retaining 8 out of the 12 interactions detected at ZT6. Because chromatin interactions shape transcription [56, 57], we sought to explore the gene content and their transcriptional dynamics within the novel contacts found in livers from obese mice. Three interactions were significantly increased at ZT6 and five at ZT18 in these livers (Fig. 2B, C). At ZT6, *Dbp* in livers from obese mice established new contacts with genes *Bcl2l12* (BCL2-Like 12), *Irf3* and *Fgf21*, and at ZT18, new contacts included *Akt1s1*, *Pnkp* (Polynucleotide Kinase 3’-Phosphatase), *Rpl13a*/*Flt13l* (ribosomal protein L13a), *Bcat2* (Branched Chain Amino Acid Transaminase 2), *Fgf21* and *Grwd1* (Glutamate Rich WD Repeat Containing 1). Notably, several of these genes are related to the AKT pathway, involved in diet-induced insulin resistance and hepatic steatosis [58]. For example, both FGF21 and AKT1S1 (also known as PRAS40) are substrates for phosphorylation by AKT kinase [59, 60], and effect fatty liver disease, while IRF3 mediates HFD-induced insulin resistance and impaired hepatic glucose metabolism trough a mechanism involving AKT [61]. Accordingly, gene expression analyses showed that these genes were overexpressed in livers from obese mice either at the mRNA or at the pre-mRNA level (Fig. 2D). Differently, expression of *Rpl13a* or small nuclear RNA genes which also establish new contacts with *Dbp* under high fat conditions, remained unaltered in livers from obese mice (Figure S3A). Hereby, contacts between *Dbp* and distal genes in fatty mouse livers conform an interacting cluster sustaining expression of metabolic genes involved in the NAFLD phenotype. In obesity, rhythmic contacts with the *Dbp locus* were underrepresented, appearing more stable contacts which favor a rearrangement of chromatin loops allowing cyclic *Dbp* expression while coordinating transcription of HFD-responsive genes.

Extensive transcriptional alterations are triggered by high-fat diet, including reprogramming of rhythmic transcription. Hereby, we sought to study the network of long-range interactions involving regulatory regions of the genes *Ppar*γ, *Ppar*α and *Srebp1c,* largely responsible for such transcriptional responses [21, 40]. In this context, we reasoned that the remodeling of genomic regulatory elements governing the transcriptional activation of these key genes during high-fat feeding might initiate the transcriptional responses to high fat feeding.

*Ppar*γ gene encodes two isoforms, *Ppar*γ1 and *Ppar*γ2, using different promoters and alternative splicing [62]. Under high fat feeding, PPARγ2 protein is overexpressed in hepatocytes promoting the adipogenic program to store fatty acids in lipid droplets [62–64] (Fig. 2E). This is accompanied by *Ppar*γ2 overexpression at the mRNA and pre-mRNA levels, evidencing transcriptional control (Fig. 2F). Consistently, *Pparγ2* promoter engaged in multiple long-range interactions in the liver of high-fat diet fed mice, both at ZT6 and ZT18 (Figs. 2G, 2H). Most of these (6 out of 8) were conserved between both ZTs, which appeared within gene bodies including *Tsen2* (TRNA Splicing Endonuclease Subunit 2), *Timp4* (Tissue inhibitor of metalloproteinases 4) and *Gm17482*. We explored whether the diet-induced chromatin contacts influenced transcription of these genes, and found that overall, their expression remained unchanged independently of ZT or diet (Fig. 2I).

**Fig. 3:**
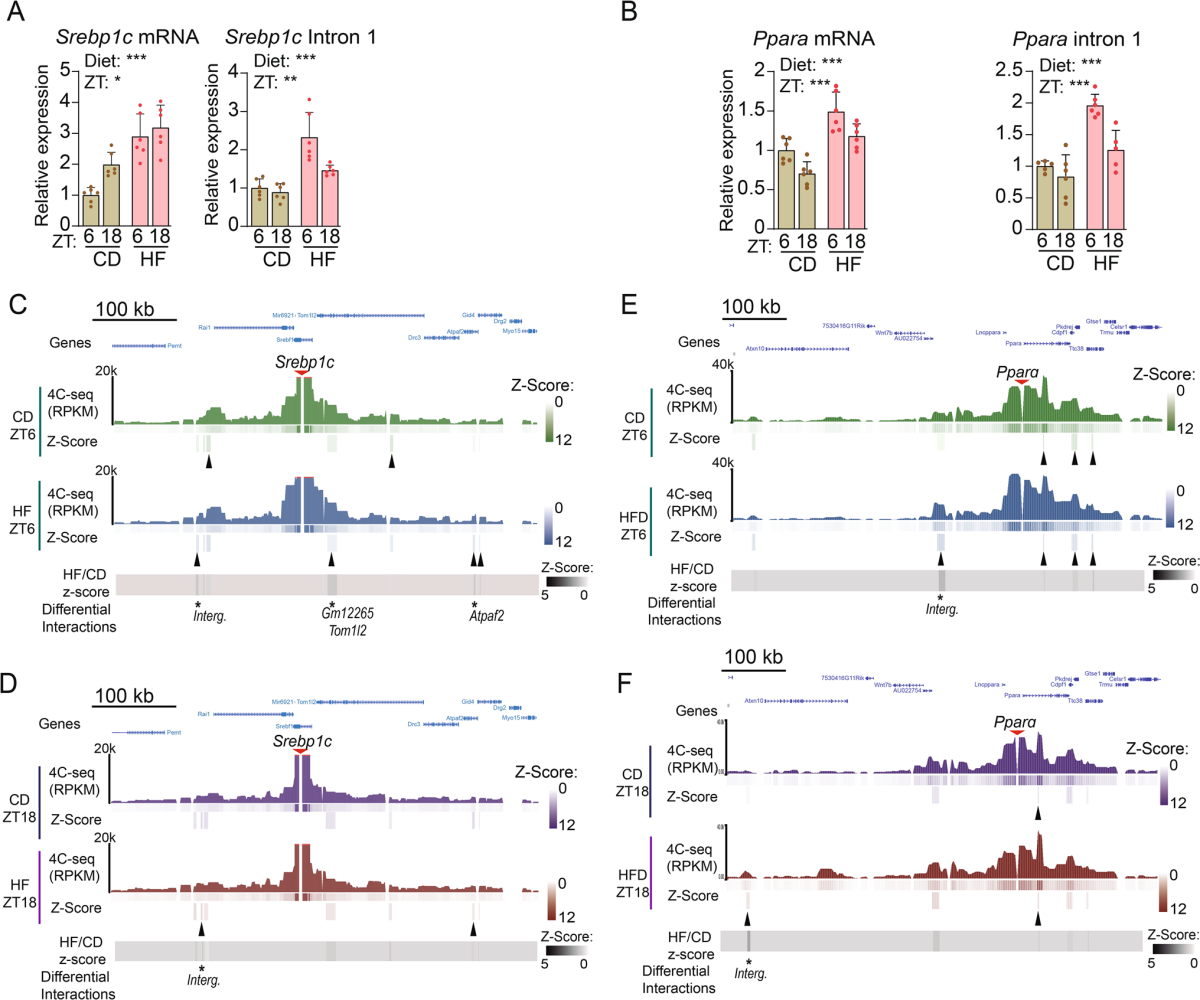
High-fat diet promotes long-range chromatin interactions around metabolic genes depending on time-of-day. **A**, **B** RT-qPCR of mature (*Srebp1c* mRNA (A), *Ppara* mRNA (B)) or nascent (*Srebp1c* intron (A), *Ppara* intron (B)) transcripts from mouse liver fed either a control (CD) or a high fat (HF) diet, at two circadian times (ZT6 and ZT18). n = 5–6 biological replicates. **C**, **D** *Srebp1c* interactome in a 1 Mb genomic window in CD and HF groups at ZT6 (**C**) and ZT18 (**D**). Interactions with *z*-score ≥ 1.5 and FDR ≤ 0.05 are indicated as black arrowheads. Differential contacts between HF and CD with a (HF/CD *z*-score rate ≥ 1.5) are indicated with *. Genes allocated within contacts are shown. **E**, **F** *Ppara* interactome in a 1 Mb genomic window in CD and HF groups at ZT6 (**E**) and ZT18 (**F**). Interactions with *z*-score ≥ 2 and FDR ≤ 0.05 are indicated as black arrowheads. Differential contacts between HF and CD with a (HF/CD *z*-score rate ≥ 1.5) are indicated with *. Genes allocated within contacts are shown. For RT-qPCR analyses, expression of *B2m* and *Tbp* housekeeping genes was used to normalize, and data from CD at ZT6 was set to 1. Two-way ANOVA was applied for statistical analyses. *p < 0.05, **p < 0.01; ***p < 0.001. Histograms represent mean ± standard error. Interg.: Intergenic region

A similar case was found for the interactions engaged by the *Srebp1c* and *Pparα* promoter loci. Hepatic SREBP1 and PPARα expression present robust daily rhythms in obese mice, and act in concert to promote rhythms in de novo lipogenesis and fatty acid oxidation (FAO) [21, 34]. Accordingly, we found overexpressed *Srebp1c* and *Ppar*α transcripts at both the mRNA and pre-mRNA levels, after high fat feeding (Fig. 3A, B). In both cases, the pre-mRNA was significantly overexpressed at ZT6. Our 4C-seq experiments using regulatory regions of these genes as baits also revealed coherent and significant differences between the day and night interactomes. Specifically, for *Srepb1c*, we found 2 significant interactions in control mice and 4 in obese mice at ZT6, while at ZT18, these were reduced to none and two interactions respectively, in concordance with reduced transcription at this ZT (Fig. 3C, D). Concomitantly, *Pparα* promoter engaged with 3 distant regions in the liver of control diet fed mice, and 4 in obese mice at ZT6. At ZT18, these were reduced to one and 2 long-range interactions respectively (Fig. 3E, F).

Collectively, these findings sustain the notion that a high-fat diet promotes precise reconfigurations of chromatin contacts within the regulatory elements essential for metabolic adaptations to dietary changes. Notably, specific contacts exhibit dependency on the time of day, and appear concurrent with fluctuations in the transcriptional activity of these pivotal genes, highlighting the interplay between chromatin architecture and the temporal regulation of gene expression in response to a high-fat diet.

### Regulatory regions in fatty liver-specific interactions acquire marks of transcriptional activity and are bound by circadian and lipid response regulators

**Fig. 4:**
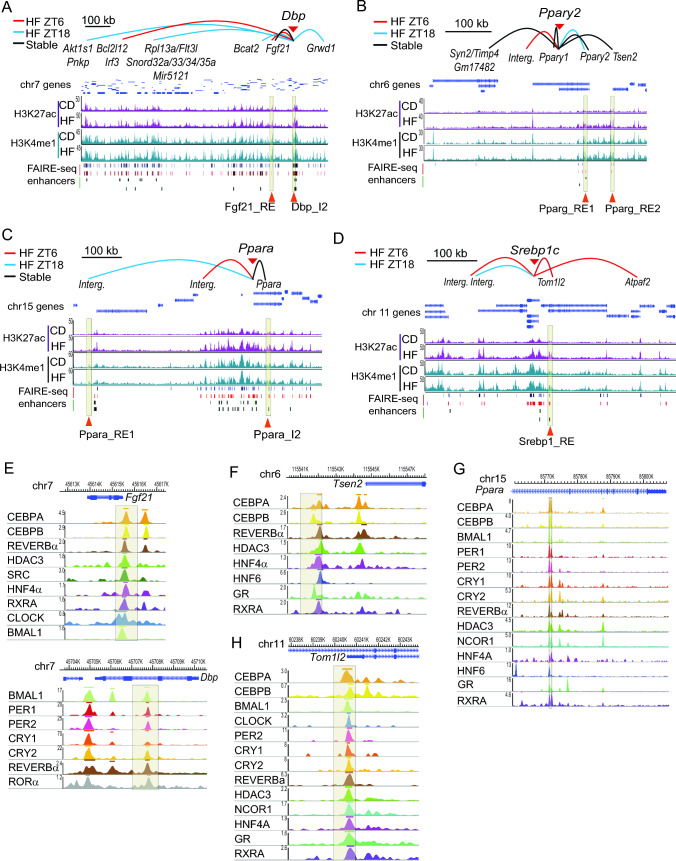
Time- and diet-dependent interactions between regulatory elements within a cistrome modulated by the molecular clock. **A**–**D** Spider plots show hepatic chromatin interactions triggered by high fat feeding at ZT6 (red), ZT18 (blue) or both times (black) engaged by *Dbp* (**A**), *Pparg2* (**B**), *Ppara* (**C**) and *Srebp1c* (**D**) 4C baits, including genes allocated within these interactions. Underneath, the corresponding genome browser view of H3K27ac (purple) and H3K4me1 (green) occupancy for each genomic region is shown, from livers of mice fed either a control (CD) or a high fat (HF) diet. Below, regulatory regions uncovered by FAIRE-seq from livers of mice fed either a control (blue) or a high fat (red) diet are indicated, followed by predicted enhancers. Genomic regions highlighted within a yellow box indicate selected enhancer elements. **E**, **F** Genome browser view of the indicated transcription factors to the regulatory regions (**E**) *Fgf21*_RE (top) and DbpI2 (bottom), (**F**) *Pparg*_RE2, (G) *Ppara*_I2 and (H) *Srepb1*_RE

Given the functional implications of long-range interactions in bringing distant regulatory elements into proximity, we sought to explore the regulatory potential inherent in the identified chromatin contacts [50]. To do so, we obtained from databases high-resolution genome-wide distribution maps of monomethylated histone H3 lysine 4 (H3K4me1) and acetylated histone H3 lysine 27 (H3K27ac) [65, 66] within the livers of both lean and obese mice. These specific histone marks serve as markers for regulatory elements acting as transcriptional enhancers, and the enrichment of H3K27ac indicates increased enhancer activity. Concurrently, chromatin accessibility was profiled in the genome using FAIRE-seq data [67], together with previously described enhancer elements [9, 21] (Fig. 4A–D). Our study revealed a notable increase in H3K27ac in the vicinity of the of *Pparg*, *Ppara* and *Srebp1* genes in the livers of obese mice suggesting the likely involvement of these regions in the upregulation of these genes in response to high-fat feeding (Fig. 4B–D). In contrast, the *Dbp* locus, situated within a markedly gene-rich region, did not exhibit a pronounced enrichment of the H3K27ac mark, except for discrete loci (Fig. 4A). Specifically, a newly interacting region in livers of obese mice encompassing the *Fgf21* gene (Fig. 4A, *Fgf21*_RE), and the *Dbp* intron 2 (*Dbp*_I2), both displayed elevated H3K27ac in livers from obese mice. These findings suggest a potential role for these regions as enhancer elements orchestrating genomic responses to high-fat feeding in a time-of-day dependent manner.

In addition, we observed specific distal interactions with the *Ppar*γ bait which appeared in response to high fat feeding and were situated within a genomic region exhibiting a notable increase in the H3K27ac mark in the livers of obese mice. These identified regulatory elements were designated as *Pparg*_RE1 and *Pparg*_RE2 (Fig. 4B). A parallel pattern emerged for the genomic locus housing the *Pparα* gene, revealing two contacts enriched with H3K27ac. These included a distal upstream region labeled as *Ppara*_RE1 and a contact within the *Pparα* intron 1 designated as *Ppara*_I2 (Fig. 4C). Lastly, a similar distinctive interaction was selected upstream of the *Srebp1c* gene, denoted as *Srebp1*_RE (Fig. 4D). This pattern of specific distal interactions, coupled with the distinctive H3K27ac enrichment, underscores potential regulatory roles for these elements in the context of high-fat feeding-induced genomic responses.

To validate the potential responsiveness of the selected genomic regions to specific transcriptional regulators in the context of high-fat feeding, we conducted an unbiased analysis utilizing the Cistrome Database toolkit [29]. This analysis involved a comprehensive search for potential transcriptional regulators enriched in these regions, leveraging experimental ChIP-seq data from mouse liver and adipose tissues [68, 69]. Our analyses revealed that these regulatory elements exhibit the capacity to recruit a diverse array of transcription factors associated with the clock machinery, such as BMAL1, CLOCK, REVERBα, as well as hepatic and lipid-responsive transcription factors including CEBPβ, HNF4A (hepatocyte nuclear factor 4 alpha), GR (glucocorticoid receptor), RXRA/G (retinoid X receptor), PPARγ2, and PPARα. Additionally, our results implicated epigenetic regulators, such as KTM2B (lysine methyltransferase 2B), ARID1A (AT-rich interactive domain-containing protein 1A), SMARCA4 (SWI/SNF related, matrix associated, actin dependent regulator of chromatin, subfamily A, member 4), or HDAC3. Notably, specific for the *Ppara*_RE locus, the recruitment of proteins involved in supporting genome architecture, including CTCF (CCCTC-Binding Factor), RAD21 (RAD21 cohesin complex component), MED1, or BRD4 (bromodomain-containing protein 4), was evident (Figure S4A–D). When visualizing ChIP-seq data from mouse liver for the circadian components, it was evident that the selected loci could recruit the clock machinery (Fig. 4E–G). Interestingly, the contacts Fgf21_RE, Pparg_RE2, Ppara_I2 and SREBP1_RE were bound by both transcriptional activators of the CEBP family, and the co-repressor complex formed by NCoR and HDAC3 (Fig. 4E–G). Notably, NCoR and HDAC3 are recruited to specific loci by REVERBα to regulate rhythmic hepatic lipid metabolism [70, 71], while CEBP proteins are well-known drivers of adipogenesis and have been extensively implicated in the control of hepatic lipid homeostasis [72, 73]. Together, these data suggest that under high fat feeding, new long-range interactions appear in concordance with transcriptional responses, which might be mediated by components of the circadian machinery and specific lipid-responsive transcription factors.

### CEBPβ counteracts REVERBα activity to promote lipid-responsive enhancer function

**Fig. 5:**
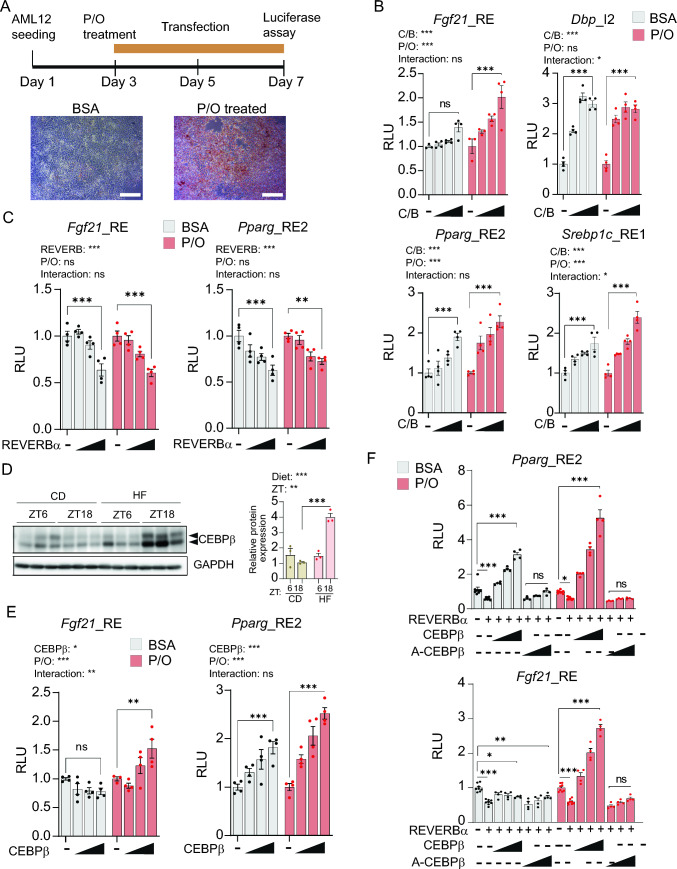
Lipid-responsive enhancers activated by CEBPβ by obstructing REVERBa repressor function. **A** Experimental set-up of AML12 hepatocyte cells treatment with 0.25 mM 1:2 palmitate/oleate (P/O), and subsequent transfection (top). Representative images of ORO staining of AML12 cells treated with Bovine Serum Albumin (BSA, control) or P/O. White bars correspond to 100 μm. **B**, **C** Luciferase-based enhancer activity assays with the selected enhancer regions in AML12 cells transiently transfected with increasing amounts of plasmids encoding (**B**) CLOCK/BMAL (C/B, 50, 100, 200 ng each) or (**C**) REVERBα (100, 200, 400 ng), in the presence or absence of lipids (P/O). **D** CEBPβ protein expression in mouse liver fed either control (CD) or high fat (HF) diet was assessed by western blot at two circadian time points (ZT6 and ZT18). Histograms represent quantification of 3 independent biological replicates, normalized to GAPDH. (means ± s.e.m.; *p ≤ 0.05, **p ≤ 0.01, ***p ≤ 0.001; Two-way ANOVA with Bonferroni´s post-test). **E** Luciferase-based enhancer activity assays with the indicated enhancers in AML12 cells transiently transfected with increasing amounts of plasmid encoding CEBPβ (100, 200, 400 ng) in the presence or absence of lipids (P/O). **F** Luciferase-based enhancer activity assays with the indicated enhancers in AML12 cells transiently transfected with the indicated plasmids. Increasing amounts of plasmids expressing CEBPβ or A-CEBPβ (100, 200, 400 ng) were assessed, in the presence or absence of lipids (P/O), and 400 ng of REVERBα encoding plasmid. For all luciferase assays, light units were normalized to an internal LacZ control, and the relative light units (RLU) from basal expression was set to 1 (means ± s.e.m. of 4 replicates). Two-way ANOVA and Bonferroni´s post-test was used. *p ≤ 0.05, **p ≤ 0.01, ***p ≤ 0.001, ns: not significant

To corroborate the hypothesis that the molecular clock might promote the activity of specific enhancers in the liver in response to lipid accumulation, we established a cellular system using the hepatocyte AML12 cell line to induce intracellular lipid accumulation (Fig. 5A). After three days of treatment with a mixture of palmitic and oleic acids (P/O), most cells exhibited detectable intracellular lipids (Fig. 5A). Concomitantly, to test the transactivation potential of the transcription factors, the selected genomic contacts were cloned into a luciferase reporter enhancer assay vector. Additionally, a non-related loci was similarly cloned to serve as a negative control for basal luciferase expression (NR-Luc), while a previously described plasmid expressing the luciferase gene under the control of *Dbp* promoter (*Dbp*-Luc) was used as a positive control [32]. As expected, CLOCK/BMAL1 overexpression in AML12 cells increased *Dbp* promoter activity but did not induce the NR-Luc expression (Figure S5A). Likewise, the *Dbp*_I2 genomic region, containing two e-boxes known to strongly drive rhythmic *Dbp* expression [55], exhibited high responsiveness to CLOCK/BMAL1 (Figure S5B). Among the other contacts, *Fgf21*_RE, *Pparg*_RE2 and *Srebp1c*_RE1 displayed robust enhancer activity in the presence of CLOCK/BMAL1 (Figure S5C). Distinctly, the *Pparg*_RE1 contact, which was preferably bound by structural proteins (Figure S4B), did not show significant enhancer activity (Figure S5C). Conversely, both *Ppara*_RE1 and *Ppara*_I2 loci displayed strong enhancer activity which appeared unaffected by the presence of CLOCK/BMAL1 proteins (Figure S5D). To gain insights into the influence of lipids on the enhancer potential of these CLOCK/BMAL1-responsive enhancers, increasing expression of CLOCK/BMAL1 was evaluated in the presence or absence of P/O (Fig. 5B). Notably, the three enhancer regions *Fgf21*_RE, *Pparg*_RE2 and *Srebp1c*_RE showed increased CLOCK/BMAL1-dependent activity when cells where exposed to P/O (Fig. 5B p < 0,001, Two-way ANOVA with Tukey post-test). Additionally, increased expression of the repressor REVERBα opposed the enhancer activity of *Fgf21*_RE and *Pparg*_RE2 in a dose-dependent manner to a similar extent in the presence or absence of lipids (Fig. 5C). These findings reveal an interplay between the molecular clock, lipid accumulation, and the dynamic regulatory activity of specific enhancers in liver cells.

**Fig. 6:**
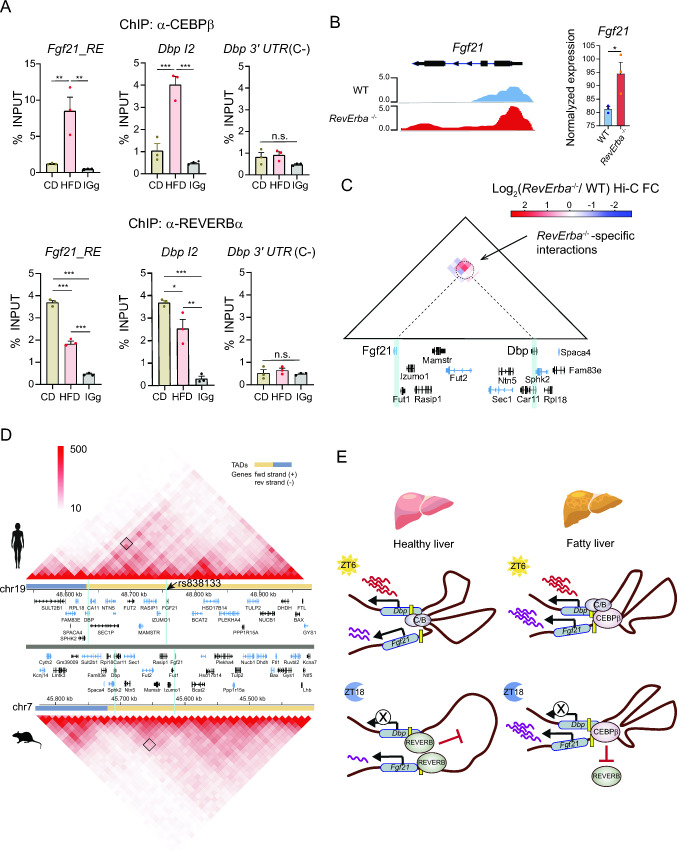
Comparative genome organization and proposed regulatory mechanism for DBP_I2 and FGF21_RE enhancers **A** ChIP experiments followed by qPCR were performed in livers from CD and HF diets fed mice at ZT10, corresponding to the time when REVERBα is highly recruited to chromatin, using the indicated antibodies*.* A *Dbp* 3’UTR region was assessed as a negative control locus. n = 3 biological and 2 technical replicates. (means ± s.e.m.; *p ≤ 0.05, **p ≤ 0.01, ***p ≤ 0.001; Two-way ANOVA with Bonferroni´s post-test). **B** (left) Global Run-On seq (GRO-seq) from livers of WT and liver-specific *Rev-erbα* ^−/−^ mice at ZT10 is shown as normalized counts (RPKM). (Right) *Fgf21* mRNA expression as detected by microarray from livers of WT and liver-specific *Rev-erbα* ^−/−^ mice at ZT10.. (mean ± s.e.m.; *p ≤ 0.05, Student’s *t*-test) (**C**) Differential Hi-C analysis at the *Fgf21* and *Dbp* locus revealing *Rev-erb*α KO-specific interactions at ZT10, represented as log_2_ ratio (ZT10 Rev-erbα KO Hi-C/ZT10 WT Hi-C). **D** Evolutionary conservation of the genome organization between mouse and human at the region allocating *Dbp* an *Fgf21* genes**.** HiC data from human hepatic HepG2 cell line (Top, ENCODE 3 realease [92]), and mouse embryonic stem cells (bottom) [93] are visualized in the 3D Genome browser using the Compare HiC application (http://3dgenome.org) [94]. Heatmaps, representing interaction frequencies at a 10 Kb resolution, demonstrate higher interaction frequencies in redder regions. TADs are indicated at the bottom, with blue vertical lines denoting the positions of Dbp_I2 and FGF21_RE regions. Black squares in the heatmaps signify interaction frequencies between the two loci. The *FGF21* variant rs838133 resides within the FGF21_RE locus in the promoter of *FGF21* and is indicated with a black arrow. Note that the mapped region of mouse chromosome 19 is inverted to mirror the position of human genes. **E** Proposed model for the mechanism regulating the interaction between *Dbp*_I2 and *Fgf21*_RE. In healthy conditions, *Dbp* undergoes circadian transcription during the day and is silenced at night through canonical clock machinery action. Additionally, a dynamic circadian interactome shows increased genomic contacts during the day and fewer at night. In fatty livers, *Dbp* transcriptional dynamics mimic those of a healthy liver. However, the interactome is influenced by lipid-responsive transcription factors, particularly CEBPβ, promoting interactions between the regulatory element Dbp_I2 and the distant obesity-associated hepatokine *Fgf21*. During the day, CLOCK/BMAL1 permits DBP expression, while at night, CEBPβ counters REVERBα repressor activity, maintaining *Fgf21* expression and increasing contacts around the DBP_I2 enhancer

Recent research has underscored the involvement of CEBPβ in hepatic rhythmic gene expression, particularly in collaboration with BMAL1 on specific genomic regulatory elements. Intriguingly, this molecular interplay is heavily influenced by food intake [74]. Additionally, it has been postulated that long-range interactions between regulatory elements underlies tissue-specific circadian transcription [12]. Notably, CEBPβ appeared overexpressed in the liver of obese mice, specifically at ZT18 (Fig. 5D  p< 0.001, Two-way ANOVA with Tukey’s post-test). In this context, given the consistent recruitment of CEBPβ to specific hepatic chromatin interactions in obese mice (Figs. 4E–H, S4), we aimed to investigate whether CEBPβ could regulate the enhancer activity of these genomic contacts. Our findings reveled that CEBPβ increases the enhancer activity of Fgf21_RE specifically in the presence of lipids (Fig. 5E). In the case of Pparg_RE2, CEBPβ promoted the enhancer activity in a dose-dependent manner both in the presence and absence of lipids, with a significantly higher effect observed in P/O-treated cells (Fig. 5E; p < 0.001, Two-way ANOVA with Bonferroni post-test). Because REVERBα is a general inhibitor of enhancer-promoter loop formation in rhythmically transcribed genes [14], we wondered if CEBPβ could modify this mechanism. Coexpressing REVERBα with increasing amounts of CEBPβ revealed that CEBPβ hinders REVERBα-mediated inhibition of the enhancer function of *Fgf21*_RE in a dose-dependent manner specifically in the presence of lipids (Fig. 5F  p< 0.001, Two-way ANOVA with Bonferroni post-test). Similarly, CEBPβ potentiated the enhancer function of *Pparg*_RE2 counteracting the REVERBα activity with a more pronounced effect in lipid-treated cells (Fig. 5F). In contrast, a dominant negative version of CEBPβ lacking functional DNA-binding and transactivation domains (A-CEBPβ) [75, 76] did not influence *Fgf21*_RE or *Pparg*_RE2 enhancer function (Fig. 5F). To further confirm this lipid-dependent crosstalk between REVERBα and CEBPβ in vivo, we performed ChIP experiments in mouse livers. Under high fat diet conditions, CEBPβ showed increased recruitment to chromatin at the *Fgf21*_RE, *Dbp*_I2 and *Pparg*_RE2 loci, while REVERBα binding appeared significantly lost when compared with the control livers (Fig. 6A, S5E). Additionally, we analyzed available gene expression data from liver-specific *Rev-erba*^−/−^ mice [9], showing that in these mice, hepatic nascent and mature *Fgf21* transcriptional levels were increased (Fig. 6B). This was coherent with increased interactions between the *Fgf21* and *Dbp* loci in livers from *Rev-erba*^−/−^ mice (Fig. 6C), as visualized by differential analysis of available HiC data [14].

These data unveil a role for CEBPβ in delineating the three-dimensional chromatin landscape in the liver of obese mice, sustaining a transcriptional program oriented to adjust to dietary conditions in collaboration with the clock machinery.

## Discussion

The circadian system organizes physiology around the day, anticipating daily environmental fluctuations. The molecular gears governing circadian rhythms act in concert on chromatin to coordinate transcriptional oscillations of thousands of genes, which are involved in critical cellular pathways, as a primordial step to trigger physiological circadian outputs. Transcriptional regulation of rhythmic metabolic pathways results from the tight collaboration between the molecular clock and tissue-specific transcription factors both in health and disease [77]. Concomitantly, chromatin structure itself also conforms a permissive or repressive role contributing to circadian transcriptional regulation through physically connecting regulatory elements in the genome in a tissue-specific manner [78]. Rhythmic chromatin interactions have been extensively and thoroughly studied in mouse liver, evidencing that enchancer-promoter interactions with varied dynamics around the day underlie circadian transcription [14, 15]. However, less is understood on how genome structures impact cyclic gene expression in disease. Here, we analyzed the genome interactions in livers from lean and obese adult mice at two circadian time points, ZT6 (rest phase) and ZT18 (active phase) and show that diet- and time-dependent genomic interactions sustain expression of target genes.

We described that activation of novel enhancer/promoter loops accompanies transcriptional responses to lipid-rich diet in the liver. The case of *Dbp* is paradigmatic, because is located within a gene-dense chromosome region, and upon high-fat exposure, chromatin reorganization occurs to pressure gene co-expression inside the TAD. This is accompanied by activation of genes involved in insulin signaling, including *Akt1s1, Irf3* and *Fgf21* [59–61]. This suggests that these changes may facilitate metabolic flexibility, enabling the liver to better manage the increased lipid load associated with a high-fat diet. Notably, FGF21 is a hepatokine which has metabolic effects, including increased hepatic fatty acid oxidation and ketogenesis in obese mice [79, 80]. Aside from liver, FGF21 acts primarily in adipocytes, increasing glucose uptake and mitochondrial oxidative capacity, and promoting adiponectin production [81, 82]. In mice and humans, FGF21 appears cardioprotective, counteracting for example cardiac hypertrophy and diabetic cardiomyopathy disease [83–85]. Given its therapeutic potential, analogues of FGF21 are currently undergoing clinical trials for the treatment of steatohepatitis [86]. We show that a regulatory region in the promoter of *Fgf21* identified as *Fgf21*_RE, and a regulatory element located 90 Kb upstream within Intron 2 of the *Dbp* gene, named *Dbp*_I2, strongly interact in fatty livers. This is accompanied by increased levels of H3K27ac and *Fgf21* expression, suggesting that this chromatin arrangement is functional [87]. Mouse *Fgf21*_RE sequence is highly conserved amongst vertebrates, and in humans, this region shows 71% of identical sequence with a locus within the *FGF21* promoter (Fig. 6D). Interestingly, the minor allele rs838133 located at this locus is robustly associated with higher sugar and alcohol preference, triglyceride levels and body fat distribution in people [88–90], yet the mechanism remains unknown. Considering our results, it is tempting to speculate that the regulatory potential of this locus might underlie metabolic dysfunction Underscoring whether the allele rs838133 compromises this interplay could provide grounds for managing personalized FGF21-based therapies. Additionally, we have shown that CEBPβ in the presence of lipids potentiates the enhancer activity of *Fgf21*_RE, opposing REVERBα repressor activity (Fig. 5E), and in the absence of REVERBα, *Fgf21* is overexpressed and chromatin interactions between *Fgf21* promoter and *Dbp* increase (Fig. 6B, C). These data suggest that diet and time-specific chromatin loops could be part of the response to diet (Fig. 6E), and increased chromatin interactions and subsequent upregulation of *Fgf21* may serve as an adaptive mechanism to protect the cardiovascular system from the detrimental effects of excessive lipid intake. This does not exclude that in the long term, the persistent upregulation of certain pathways might contribute to metabolic derangements resultant from obesity.

Overnutrition disturbs circadian rhythms in multiple levels, including metabolic cycles and rhythmic transcription [18, 40]. In mouse liver, high-fat feeding leads to a global remodeling of circadian enhancers, imposing rhythms to enhancers neighboring genes involved in hepatic lipid metabolism. This effect is determined in part by the transcription factors SREBP1 and PPARa, while transcriptional rewiring is assisted by PPARg, the three of which become rhythmic in fatty livers [21, 40]. However, mechanisms underlying their acquired rhythmicity in response to high-fat feeding are poorly understood. Here, we demonstrate that *Srebp1c*, *Pparα* and *Pparγ2* genes reorganize their interactome in obesity to promote a chromatin architecture favoring their transcriptional activation. Increased interactions were shown for the three genes in fatty livers, yet fluctuations depending on time-of-day were also evident, presenting more interactions at ZT6. In the case of *Pparγ2,* we found a notable increase in interactions upon high-fat feeding, and some of these new contacts involved regions in close proximity or containing genes such as *Syn2, Timp4* or *Tsen2*, however, we did not detect expression changes in these genes. This might be due to several causes, including that the temporal resolution of our analysis might have missed critical time points where changes in expression could occur. Another plausible explanation is that the increased *Pparγ2* contacts observed may facilitate a poised state, making these genes more accessible for activation under specific conditions. Thus, while the regulatory elements are in closer proximity, transcriptional activation might require additional signals or cofactors. It is also possible that new chromatin contacts influence the expression of non-coding RNAs or induce epigenetic modifications that were not measured in our study, which might play crucial roles in controlling the expression of these genes. Further analyses with high temporal resolution, combined with research into other regulatory mechanisms, can provide deeper insights into the functional consequences of these chromatin interactions.

We performed an unbiased analysis of the known transcription factors that can be recruited to novel chromatin contacts in obese mice. We found colocalization of clock proteins and TFs involved in lipid metabolic control, including the CEBP family, the HDAC3-NCoR repressors, the hepatic-specific HNF4 and HNF6, or the nuclear receptors GR and RXRA. At this regard, rhythms in TF activity and tissue-specific chromatin interactions account for differences in the circadian transcriptome across organs [77, 78], but also TFs such as HNF4 and CEBPα are involved in remodeling of enhancer-promoter interactions in fatty liver [22]. Our results are coherent with this notion, where specific TFs and the clock machinery underlie differences in chromatin contacts in response to diet, while integrating time-of-day information. To test this, we selected the CEBPβ transcription factor and found that lipid-responsive enhancers could be activated by increased concentrations of CEBPβ. Importantly, the activity of the circadian repressor REVERBα was abolished by the presence of CEBPβ, suggesting that to some extent, CEBPβ can compete on selected lipid-responsive enhancers with REVERBα to preserve chromatin looping favoring expression of metabolic genes [14, 91]. Accordingly, a positive cooperation between BMAL1 and CEBPβ has been identified to regulate integration of feeding rhythms and transcriptional oscillations in the liver [74].

Understanding chromatin organization and gene regulation during the circadian cycle provides insights into how cells and organs anticipate and adapt to daily environmental fluctuations. Abnormal exposure to cues like light at night or high-fat feeding disrupts the clock system, leading to disease. Hereby, investigating chromatin and transcriptional regulation mechanisms in disease states opens therapeutic avenues to restore rhythmicity.

### Supplementary Information

Below is the link to the electronic supplementary material.

Supplementary file1 Figure S1. Physiological characterization of mice fed chow diet or high fat diet. A) Weekly weight from C57BL/6J mice fed a normocaloric (CD) or hypercaloric (HF) diet. (n= 6 per group). Two-way ANOVA and post test of Tukey * p≤0.05, ** p≤0.01, *** p≤0.001. B, C) Glucose (B, GTT) and insuline (C, ITT) tolerance tests at ZT1 and ZT13 after 10 weeks of feeeding with CD or HF diet, and their respective area under the curve (AUC) (n= 6 mice per group). One-way ANOVA and post test of Tukey * p≤0.05, ** p≤0.01, *** p≤0.001, **** p≤0.0001. AUC (area under the curve) is plotted as arbitrary units, and CD data was set to 1. D) Representative images of macroscopy visualization of livers (left), and their lipid staining with oil-red-o (ORO, right) from control (CD) and obese (HF) mice. E) RT-qPCR of from mouse liver fed either a control (CD) or a high fat (HF) diet, at two circadian times (ZT6 and ZT18) for the indicated transcripts of the clock machinery. (means ± s.e.m. of 5-6 biological replicates). F) Protein expression assessed by western blot from the indicated clock proteins in mouse liver. Quantification data is shown on the right for the three biological replicates. Data is presented as mean ± s.e.m, For BMAL1, the ratio between phosphorylated BMAL (upper signal) to total BMAL is plotted. For REVERBα and CRY1, CD at ZT6 was set to 1. For PER2, CD at ZT18 was set to 1. For histograms, Two-way ANOVA was applied for statistical analyses. When interaction was positive, Bonferroni was used as post-test. *p˂ 0.05, **p˂0.01; ***p˂0.001; ns, non-significative. Figure S2. Interchromosomal contacts engaged by the baits in the mouse liver. A-D) Circos plots representing the genome-wide view of detected inter-chromosomal interactions engaged by regulatory elements for the genes Dbp (A), Pparγ2 (B), Pparα (C), and Srebp1c (D) in livers form mice fed a chow (CD) or high-fat (HF) diets, at two circadian times, ZT6 (day) and ZT18 (night). Figure S3. Chromatin loops induced by high fat feeding do not necessarily modify expression of all their contained genes. A) RT-qPCR of from mouse liver fed either a control (CD) or a high fat (HF) diet, at two circadian times (ZT6 and ZT18) for the indicated transcripts. (means ± s.e.m. of 5-6 biological replicates). Two-way ANOVA was applied for statistical analyses. n.s.: Non significant. Figure S4. Hepatic chromatin loops induced by high fat feeding exhibit a distinctive cistrome. A-D) Cistrome toolkit analyses showing enrichment of binding sites for the transcription factors shown in the x axes, for the indicated regulatory elements for the genes Dbp (A), Pparγ (B), Pparα (C) and Srebp1c (C), uncovered in mouse liver after high-fat feeding. Figure S5. Activity of enhancers detected after high-fat feeding is boosted by CLOCK:BMAL1. A-D) Luciferase-based enhancer activity assays with the identified enhancer regions in mouse liver after high fat-feeding. AML12 cells were transiently transfected with plasmids expressing CLOCK/BMAL (C/B) in the presence or absence of lipids (P/O). Light units were normalized to an internal LacZ control, and the relative light units (RLU) from basal expression was set to 1 (means ± s.e.m. of 4 replicates). A non-regulatory region (NR) is shown as a negative control, while the previously described reporter plasmid Dbp-Luc was used as a control of the experimental set-up. E) ChIP experiments followed by qPCR were performed in livers from CD and HF diets fed mice at ZT10, corresponding to the time when REVERBα is highly recruited to chromatin, using the indicated antibodies. n=3 biological and 2 technical replicates. (means ± s.e.m.; * p≤0.05, ** p≤0.01, *** p≤0.001; Two-way ANOVA with Bonferroni´s post-test) (PDF 8657 KB)

Supplementary file2 (PDF 129 KB)

Supplementary file3 Sequencing reads per sample and mapping results. (PDF 77 KB)

Supplementary file4 Genomic coordinates of contacts (enhancers) studied from the 4C-seq data. (PDF 61 KB)

## Data Availability

The 4C-seq data generated by this study have been deposited to the NCBI Gene Expression Omnibus with accession: Series GSE254620 (token: ajodcioyhlofjmp). For public datasets, ChIP-seq data collected from public studies were obtained from GEO as follows: H3K27ac_CD, GSM1479724; H3K27ac_HFD, GSM2055367; H3K4me1_CD, GSM2055359; H3K4me1_HFD, GSM2055361; FAIRE-seq CD and HFD, GSE55581; CEBPα, GSM1037657, GSM1816821; CEBPβ, GSM1122522, GSM1111749; REV-ERBα, GSM2218847; HDAC3, GSM2406329; NCOR1, GSM647027; SRC2, GSM1280886; HNF4A, GSM2406340; RXRA, GSM541305; HNF6, GSM1463856; GR, GSM1122515; BMAL1, GSM982690; CLOCK, GSM982713; PER1, GSM982731; PER2, GSM982738; CRY1, GSM982743; CRY2, GSM982750; RORα, GSM1659692. Binding peak calls were reprocessed by the Cistrome pipeline, available at http://cistrome.org. Location of putative enhancers in livers from control and high fat diet fed mice were obtained from references [9] and [21] respectively. Gro-seq data was obtained from GEO, GSE59486, and expression data from WT and *RevErba* liver-specific KO mice was obtained from GSE59460. HiC data was obtained from GSE104129.
