# Supplementary figures and images for: Transitions in chromatin conformation shaped by fatty acids and the circadian clock underlie hepatic transcriptional reorganization in obese mice
Source: Cell Mol Life Sci. 2024 Jul 26;81(1):309. doi: 10.1007/s00018-024-05364-3 (PMC11335233; doi:10.1007/s00018-024-05364-3)

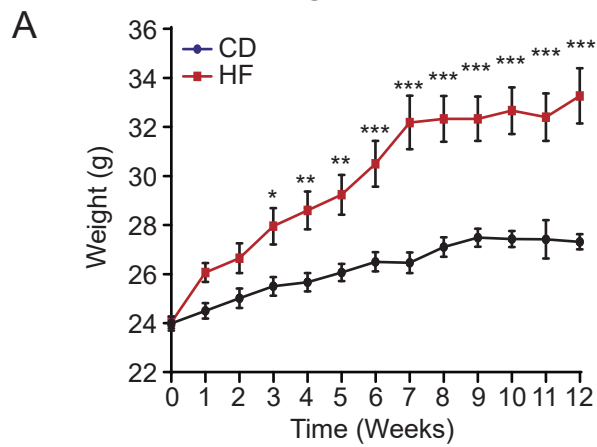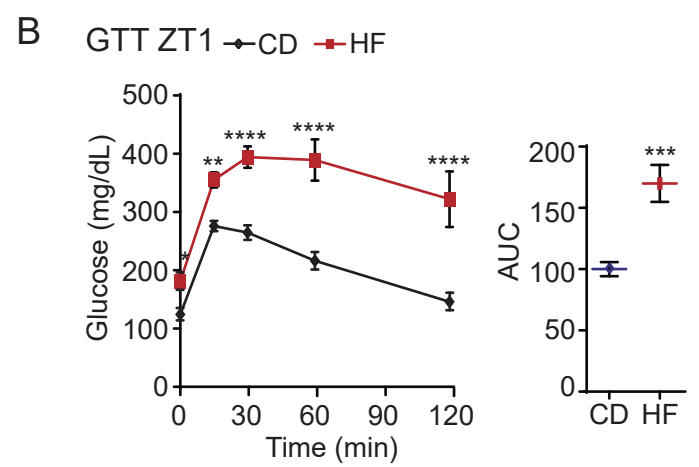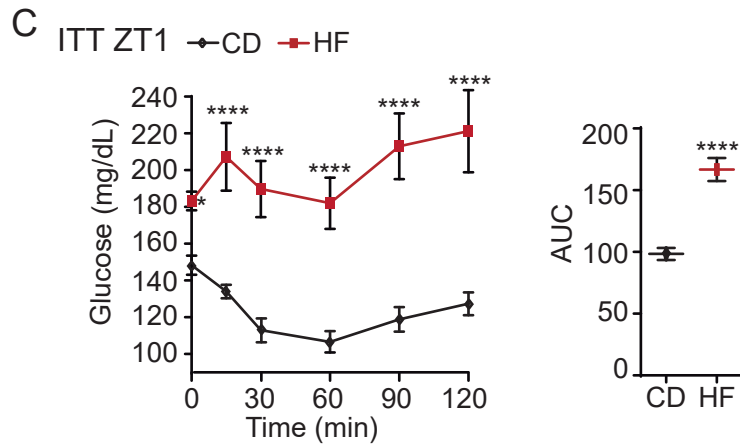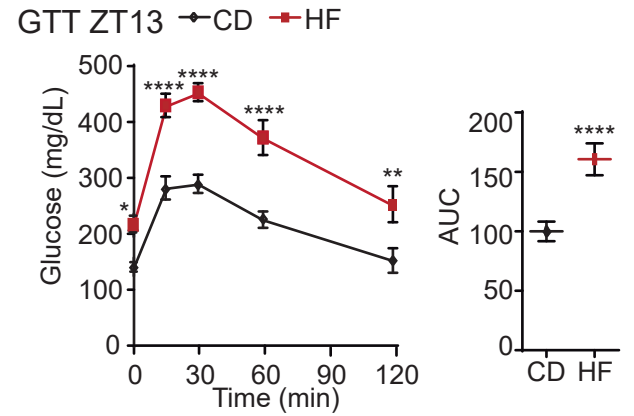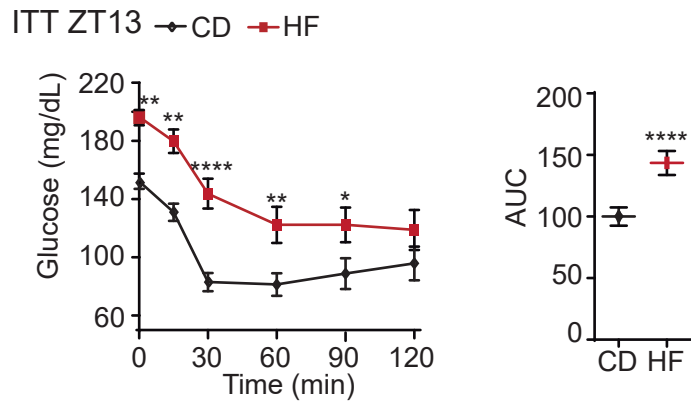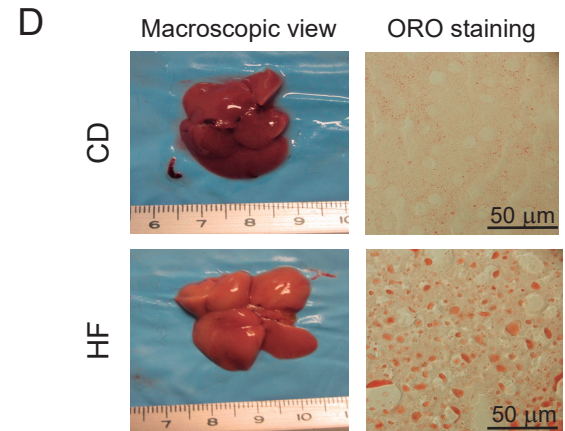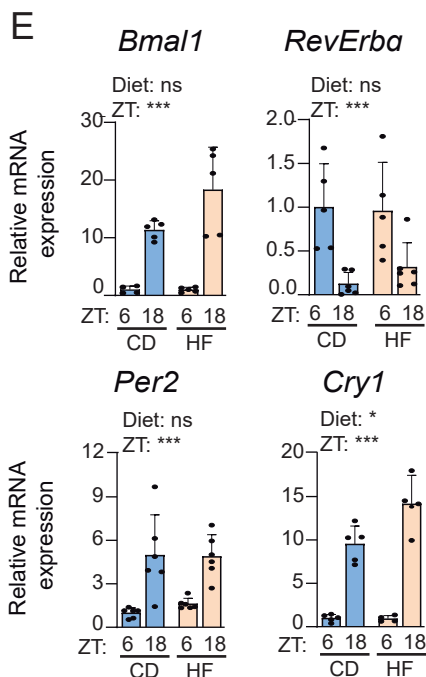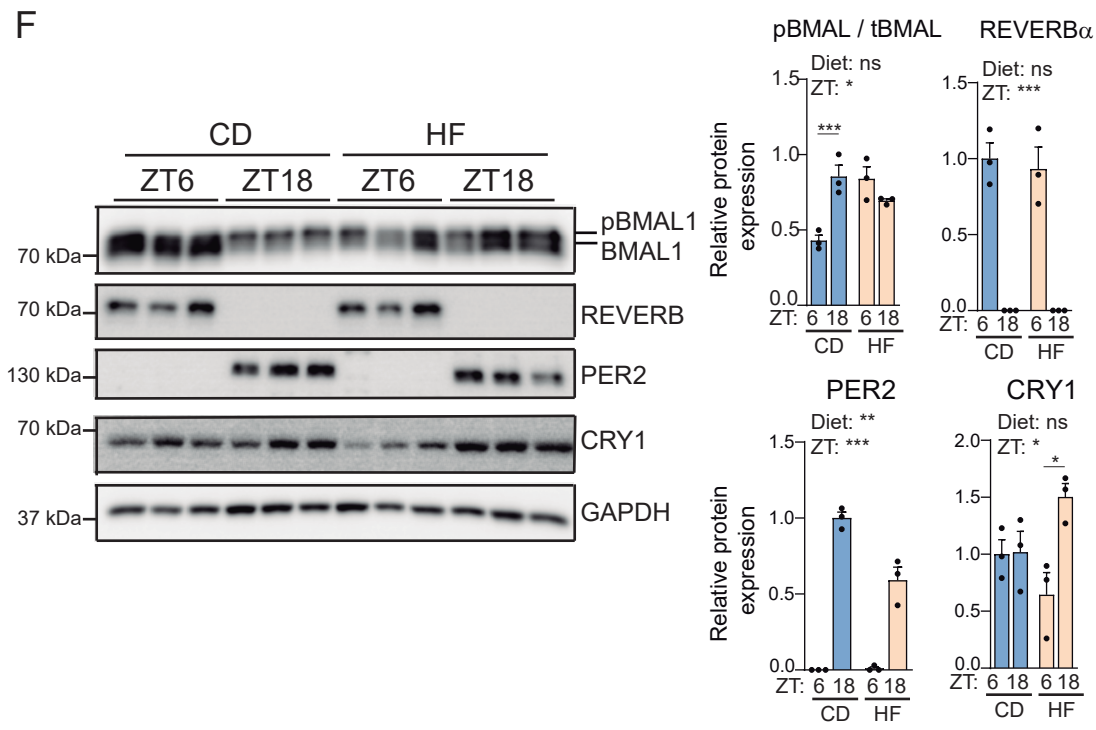

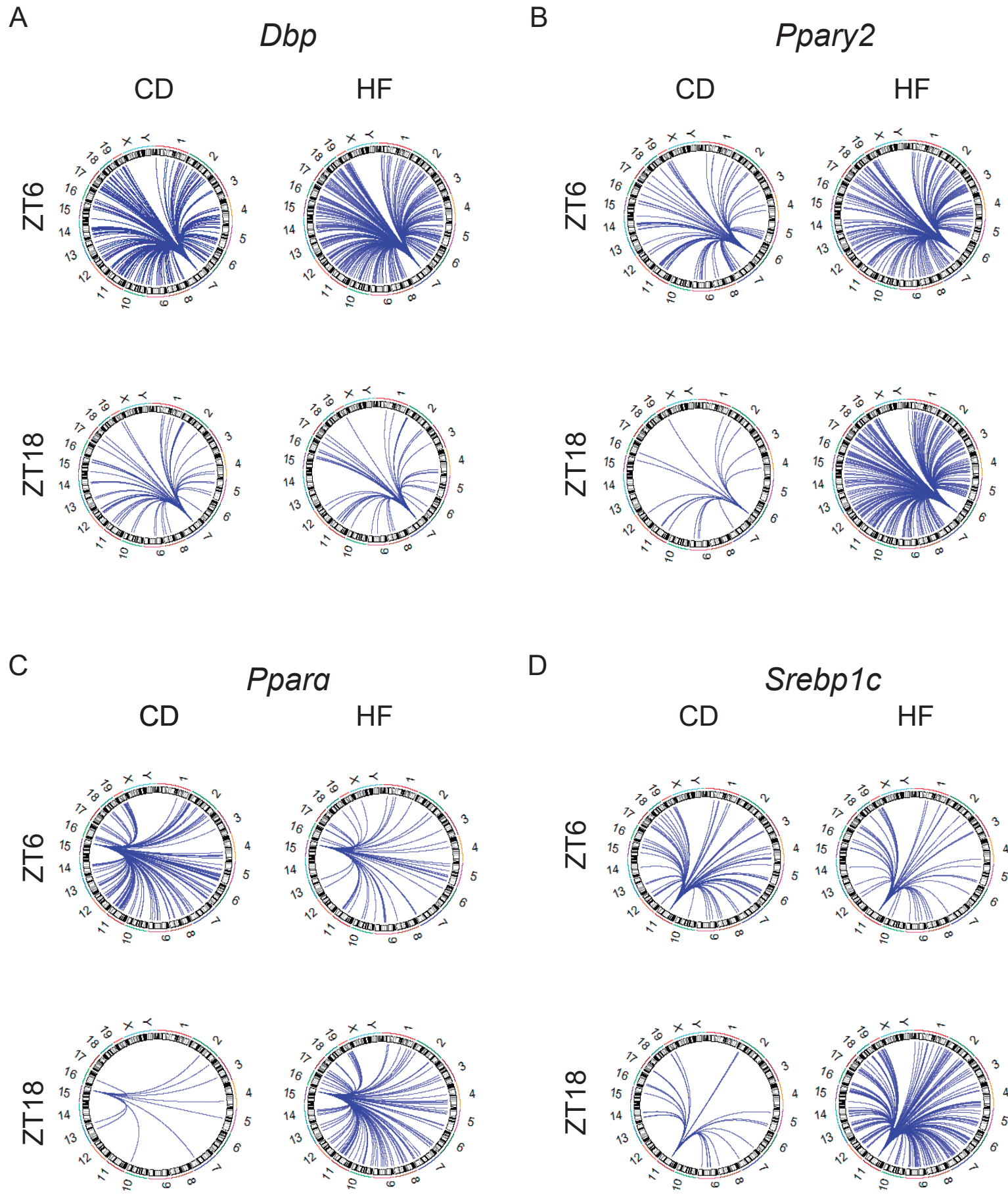

A

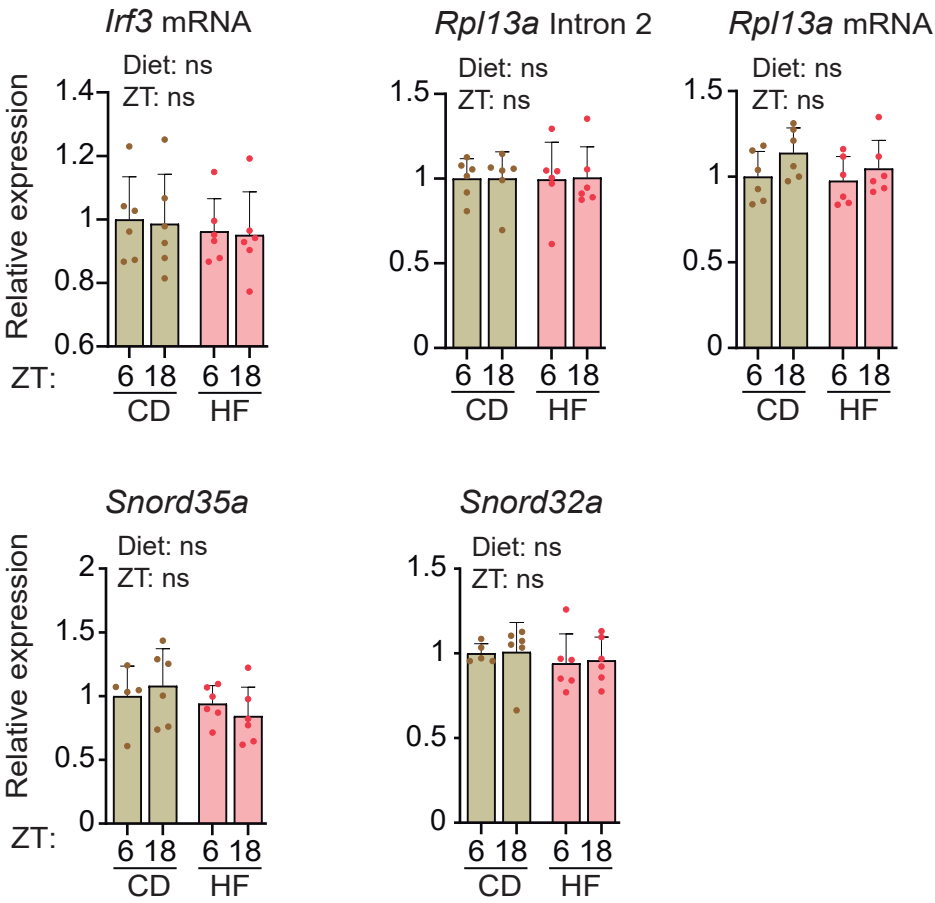

**A**
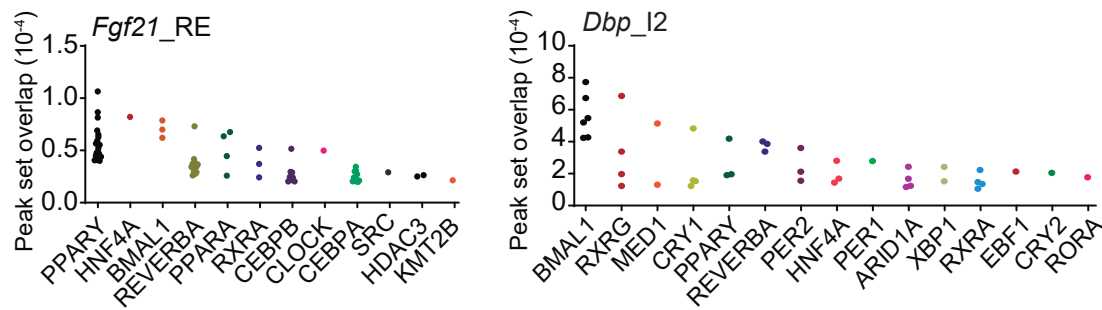
**B**
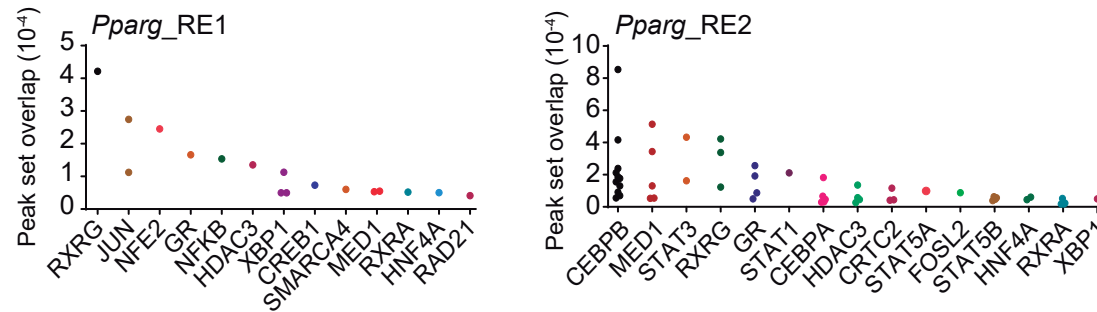
**C**
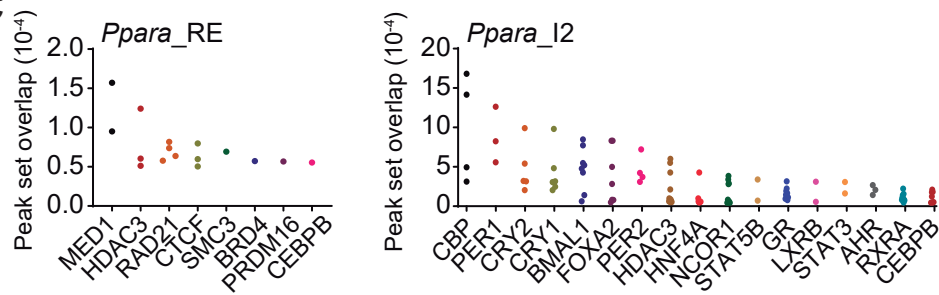
**D**
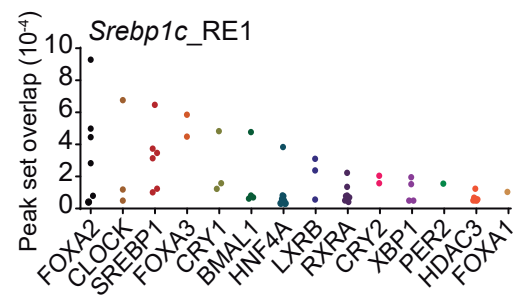

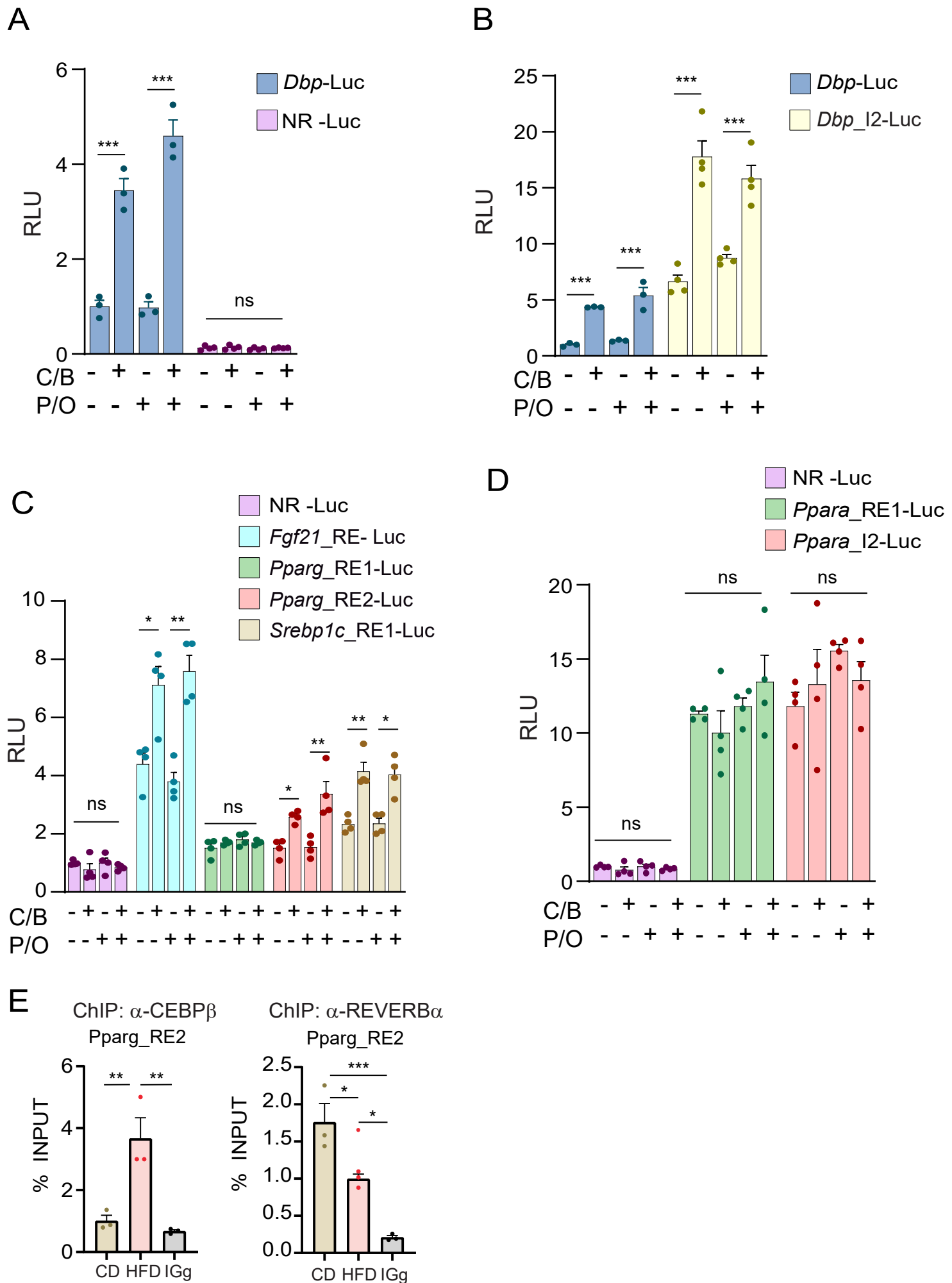

Supplement: Supplementary file 1 — Supplementary file1 Figure S1. Physiological characterization of mice fed chow diet or high fat diet. A) Weekly weight from C57BL/6J mice fed a normocaloric (CD) or hypercaloric (HF) diet. (n= 6 per group). Two-way ANOVA and post test of Tukey * p≤0.05, ** p≤0.01, *** p≤0.001. B, C) Glucose (B, GTT) and insuline (C, ITT) tolerance tests at ZT1 and ZT13 after 10 weeks of feeeding with CD or HF diet, and their respective area under the curve (AUC) (n= 6 mice per group). One-way ANOVA and post test of Tukey * p≤0.05, ** p≤0.01, *** p≤0.001, **** p≤0.0001. AUC (area under the curve) is plotted as arbitrary units, and CD data was set to 1. D) Representative images of macroscopy visualization of livers (left), and their lipid staining with oil-red-o (ORO, right) from control (CD) and obese (HF) mice. E) RT-qPCR of from mouse liver fed either a control (CD) or a high fat (HF) diet, at two circadian times (ZT6 and ZT18) for the indicated transcripts of the clock machinery. (means ± s.e.m. of 5-6 biological replicates). F) Protein expression assessed by western blot from the indicated clock proteins in mouse liver. Quantification data is shown on the right for the three biological replicates. Data is presented as mean ± s.e.m, For BMAL1, the ratio between phosphorylated BMAL (upper signal) to total BMAL is plotted. For REVERBα and CRY1, CD at ZT6 was set to 1. For PER2, CD at ZT18 was set to 1. For histograms, Two-way ANOVA was applied for statistical analyses. When interaction was positive, Bonferroni was used as post-test. *p˂ 0.05, **p˂0.01; ***p˂0.001; ns, non-significative. Figure S2. Interchromosomal contacts engaged by the baits in the mouse liver. A-D) Circos plots representing the genome-wide view of detected inter-chromosomal interactions engaged by regulatory elements for the genes Dbp (A), Pparγ2 (B), Pparα (C), and Srebp1c (D) in livers form mice fed a chow (CD) or high-fat (HF) diets, at two circadian times, ZT6 (day) and ZT18 (night). Figure S3. Chromatin loop [file 18_2024_5364_MOESM1_ESM.pdf]
