# Supplementary material for: Transitions in chromatin conformation shaped by fatty acids and the circadian clock underlie hepatic transcriptional reorganization in obese mice
Source: Cell Mol Life Sci. 2024 Jul 26;81(1):309. doi: 10.1007/s00018-024-05364-3 (PMC11335233; doi:10.1007/s00018-024-05364-3)
Supplement: Supplementary file 2 — Supplementary file2 (PDF 129 KB) [file 18_2024_5364_MOESM2_ESM.pdf]

Table S1 Primer sequences

**qPCR primers**		
**Gene**	**Forward**	**Reverse**
b2m	GGTCTTTCTGGTGCTTGTCTCA	GTTCGGCTTCCCATTCTCC
Cry1	CTGGCGTGGAAGTCATCGT	CTGTCCGCCATTGAGTTCTATG
RevErba	GGGCACAAGCAACATTACCA	CACGTCCCCACACACCTTAC
Per2	GGCTTCACCATGCCTGTTGT	GGAGTTATTTCGGAGGCAAGTGT
Bmal1	ATGCAGAACACCAAGGAAGG	CCATCCTTAGCACGGTGAGT
Pparγ1	AGAAGCGGTGAACCACTGATATTC	AGAGGTCCACAGAGCTGATTCC
Pparγ2	TGGGTGAAACTCTGGGAGATTC	AGAGGTCCACAGAGCTGATTCC
Pparγ_I1	TCTGCTGGGGATCTGAAGGC	TGTGTAAGAACTTGGGGGCAGTT
Pparγ_I6	AATCTCAGGTGGCTTTGTGGT	GTTTTTCCCAAATGCTTCCCTTAGT
Dbp	AATGACCTTTGAACCTGATCCCGCT	GCTCCAGTACTTCTCATCCTTCTGT
Dbp_I1	GTCCACGGCACCTATCAG	CTCCAGGCGTCAGATTTG
Dbp_I3	AAGGGCTCAGGTTCCAAG	GCAGAGGCAGTTCTACAGATG
Pparα	ACAAGGCCTCAGGGTACCA	GCCGAAAGAAGCCCTTACAG
Pparα_I1	CATTGCTCCTGATCGTAGTGGG	GGAGGGCAGAGACATAGGGT
Srebp1c	GGAGCCATGGATTGCACATT	GGCCCGGGAAGTCACTGT
Srebp1c_I1	CAGAGGGAAAGCAGAGGATGTGG	CCACCTTGGGCTGGAAATTGGT
Gm36355_I2	GTGGAGATCGTGTTGCCTGC	GAGACCCTGAGTTCCATTCCCAG
Syn2_I8	ACTTTCCTGGTGTGGCTGAC	CGAGCCAGTGTTTGTCTTCCA
Timp4_I2	CAAAGACCCTGCTGACACTCAA	GTTCGTCCCTCCCATAGCAC
Tsen2_I3	TTCTTTCCAGAAGCCGTCCA	GCACCAAATGAGAGCGACAC
Fgf21	TTGACACCCAGGATTTGAATGACC	GATGACGACCAAGACACTGAAGC
Fgf21_I1	CCTCCTCCTCTCAACCTCCA	GATGACGACCAAGACACTGAAGC
Fgf21_I2	TGAAACCTACGCTCTGTGAGG	GGTCATTCAAATCCTGGGTGTCAA
Akt1s1	ATGAGGACGAGCCCACTGAA	GTCTGTGCTCTCCGGGTCT
Irf_I3	CGGAAAGAAGTGTTGCGGTT	GTGGAAGTGAGCGTGTAGGT
Irf3	GCCGAACGAGGTTCAGGA	CTCTTCCAGGTTGACACGTCC
Rpl13a	CCTGCTGCTCTCAAGGTTGTTCG	CCGACCTCATGCGCCAGA
Rpl13a_I1	TGGGACACTTGCTTCATCTG	GCCATCCAATACCAGAACCTG
Snord32a	GGGAGTCCATGATGAGCAACAC	AGCGGTGCATGGGTTGAT
Snord35a	CTCAAGACTGGCACATGATGTT	CCTTTGGCATTATCGGCACTG
**Enhancer candidates**		
PPARY2_EN1	GATCGGTACCAGCTGGTTACTCTTGCCACT	GATCAAGCTTCAATCTTTCCCCCTCCCCAA
PPARY2_EN3	GATCGGTACCATGTCTCCTGTCCCCCATCA	GATCAAGCTTGGGCGGAGGAGGAAGTTTTAT
PPARY2_EN4	GATCGGTACCATGTGTTCCCTGCGTTGACA	GATCAAGCTTTCTGAGCCGTATTTCCTCCAG
Dbp_EN1	GATCGGTACCATGGCTGTTTCTCCTGTGTTGA	GATCAAGCTTCCTCAGACCAAGGAGCACAG
Dbp_EN2	GATCGGTACCGCTTTCCCATGTCCCTGTGTTG	GATCAAGCTTTGCTGTGTCCGAGTCCTACTT
Srebp1c_EN1	GATCGGTACCAGGAAGGGTCAGGGTAGCAT	GATCAAGCTTTCACCTCAGTTTGGAGCAGC
Ppara_EN1	GATCGGTACCGCAACCTCTGTATTCCCTGAAGA	GATCAAGCTTCACAGACAGCACCCCAGATA
Ppara_EN2	GATCGGTACCATGGAAGTGATAAGCCCCATCA	GATCAAGCTTGCCTCCCCTGTACCTTTACC

**4C DNA amplification primers**	
Pparγ2_EcorI	TCGTCGGCAGCGTCAGATGTGTATAAGAGACAGGGATTAGTTTTATCTCTATTTAACTTGA
Pparγ2_Csp6I	GTCTCGTGGGCTCGGAGATGTGTATAAGAGACAGATAGACTTGTTGAATAAATCACCT
Dbp_HindIII	TCGTCGGCAGCGTCAGATGTGTATAAGAGACAGAAACCTGTTCTGTGGTGGATAAGC
Dbp_Csp6I	GTCTCGTGGGCTCGGAGATGTGTATAAGAGACAGCACTGGTTTTTGCCTGGAGGTC
Pparα_HindIII	TCGTCGGCAGCGTCAGATGTGTATAAGAGACAGCATCTCCAGGGTCTCAGTTTTGCA
Pparα_Csp6I	GTCTCGTGGGCTCGGAGATGTGTATAAGAGACAGCCATTTTCCTACCCTGGCCTGTA
Srebp1c_HindIII	TCGTCGGCAGCGTCAGATGTGTATAAGAGACAGATCTGTCCCTGTCCCTTGCTGG
Srebp1c_Csp6I	GTCTCGTGGGCTCGGAGATGTGTATAAGAGACAGGCTGTCGGGGTAGCGTCTG

**ChIP**		
Fgf21 promoter	CCACTCCTGACGCGTGATATT	ATTGCATCATCCGTCCAGGC
Dbp I2	AACCAGTGCTGCACATTCCCT	CTCCTCCCCAACCAAGCACC
Dbp 3´UTR	GCCTGGAATGTATGAGCTAGCA	GGCACCGGAGTAGGCAAGA
Pparg RE2	TGTTTGTTTTTAACACTACTCTGGA	TCTGCAAATCTGCGAGGGAT
