# Supplementary material for: Transitions in chromatin conformation shaped by fatty acids and the circadian clock underlie hepatic transcriptional reorganization in obese mice
Source: Cell Mol Life Sci. 2024 Jul 26;81(1):309. doi: 10.1007/s00018-024-05364-3 (PMC11335233; doi:10.1007/s00018-024-05364-3)
Supplement: Supplementary file 3 — Supplementary file3 Sequencing reads per sample and mapping results. (PDF 77 KB) [file 18_2024_5364_MOESM3_ESM.pdf]

		MiSeq			HiSeq		
BAIT	Sample	Raw Reads (single)	Percentage of valid reads	Percentage of reads mapped to the bait´s chromosome	Raw Reads (paired)	Percentage of valid reads	Percentage of reads mapped to the bait´s chromosome
Dbp	CD_ZT6_1	527216	81.47%	58.63%	4863932	64.95%	51.60%
	CD_ZT6_2	309974	78.27%	60.64%	2750690	70.26%	61.14%
	CD_ZT18_1	242789	79.12%	56.93%	2445516	69.98%	53.58%
	CD5_ZT18_2	265276	80.66%	60.58%	2364824	64.15%	52.76%
	HF2_ZT6_1	169290	80.71%	60.15%	1644970	73.06%	60.42%
	HF4_ZT6_2	336905	82.65%	65.92%	3977224	67.18%	59.64%
	HF5_ZT18_1	358540	81%	60%	4839496	64.03%	50.50%
	HF6_ZT18_2	455179	78.83%	59.68%	4301160	62.09%	49.79%
Pparg2	CD_ZT6_1	276220	78.12%	70.96%	2566128	72.34%	90.48%
	CD_ZT6_2	347929	70.26%	68.79%	4058268	89.87%	82.38%
	CD_ZT18_1	233898	73.22%	80.58%	2821612	90.06%	90.39%
	CD5_ZT18_2	404843	75.40%	64.90%	3434648	92.63%	80.57%
	HF2_ZT6_1	134548	75.92%	74.76%	1761798	91.57%	84.10%
	HF4_ZT6_2	194853	70.38%	67.50%	1798776	89.63%	81.88%
	HF5_ZT18_1	190469	76.31%	65.93%	2057684	92.01%	79.66%
	HF6_ZT18_2	116909	71.85%	63.89%	1388736	90.87%	81.62%
Ppara	CD_ZT6_1				55998198	60.94	48.28
	CD_ZT6_2				42724176	63.79	39.43
	CD_ZT18_1				31878820	63.24	38.15
	CD5_ZT18_2				58779518	62.75	40.29
	HF2_ZT6_1				28535284	72.66	40.86
	HF4_ZT6_2				40675704	63.85	55.24
	HF5_ZT18_1				67783622	64.83	47.87
	HF6_ZT18_2				62712786	62.78	41.33
Srebp1c	CD_ZT6_1				37296356	77.22	62
	CD_ZT6_2				22332310	73.15	62.31
	CD_ZT18_1				48289422	62.43	49.73
	CD5_ZT18_2				33443614	78.8	55.32
	HF2_ZT6_1				24107190	71.21	64.51
	HF4_ZT6_2				18151738	80.11	71.62
	HF5_ZT18_1				23262378	76.73	57.63
	HF6_ZT18_2				20166186	78.68	54.67
