# Supplementary material for: Transitions in chromatin conformation shaped by fatty acids and the circadian clock underlie hepatic transcriptional reorganization in obese mice
Source: Cell Mol Life Sci. 2024 Jul 26;81(1):309. doi: 10.1007/s00018-024-05364-3 (PMC11335233; doi:10.1007/s00018-024-05364-3)
Supplement: Supplementary file 4 — Supplementary file4 Genomic coordinates of contacts (enhancers) studied from the 4C-seq data. (PDF 61 KB) [file 18_2024_5364_MOESM4_ESM.pdf]

Enhancer	mm10
Pparg_RE1	chr6:115,479,883-115,481,081
Pparg_RE2	chr6:115,540,986-115,542,182
Ffg21 _RE	chr7:45,615,136-45,616,106
Dbp_I2	chr7:45,707,006-45,708,288
Ppara_RE1	chr15:85,315,657-85,317,008
Ppara_RE2	chr15:85,771,214-85,772,193
Srebp1c_RE	chr11:60,239,955-60,240,932
